# Novel Natural Products from Extremophilic Fungi

**DOI:** 10.3390/md16060194

**Published:** 2018-06-04

**Authors:** Xuan Zhang, Shou-Jie Li, Jin-Jie Li, Zi-Zhen Liang, Chang-Qi Zhao

**Affiliations:** Gene Engineering and Biotechnology Beijing Key Laboratory, Key Laboratory of Cell Proliferation and Regulation Biology, Ministry of Education, College of Life Science, Beijing Normal University, Beijing 100875, China; zhangxuan28@outlook.com (X.Z.); LSJ19930801@163.com (S.-J.L.); lijinjie.7785004@163.com (J.-J.L.); zizhenliang@gmail.com (Z.-Z.L.)

**Keywords:** natural products, extremophilic fungi, biological activity

## Abstract

Extremophilic fungi have been found to develop unique defences to survive extremes of pressure, temperature, salinity, desiccation, and pH, leading to the biosynthesis of novel natural products with diverse biological activities. The present review focuses on new extremophilic fungal natural products published from 2005 to 2017, highlighting the chemical structures and their biological potential.

## 1. Introduction

The term “extremophile” was first proposed by MacElroy in 1974 to describe a broad group of organisms which lived optimally under extreme conditions [1], and the taxonomic range of them has been expanded from prokaryotes to all three domains—Eucarya, Bacteria, and Archaea [2]. Extremophiles are classified into seven categories according to different extreme habitats. Piezophiles reside under high hydrostatic pressure, which have been isolated from the deep-sea sediments (>3000 m depth) and the guts of bottom-dwelling animals [3,4,5]. Organisms whose optimal growth temperature ranges from 50 to 80 °C or exceeds 80 °C are called thermophiles or hyperthermophiles respectively, which have been mainly isolated from hot springs, deep-sea hydrothermal vents, and decaying plant matter [6]. Psychrophiles living in the other extreme thermal habitat have been obtained from the Antarctic, the Arctic, and glacial regions [7]. Halophiles are defined as organisms requiring >3% NaCl for growth [6]. Xerophiles thrive under the desiccated and have been discovered in ashes and deserts [8]. Acidophiles or alkaliphiles show optimal growth at pH values <4 and >9 respectively [6]. Organisms from these extreme habitats require special survival strategies for growing and reproducing, and the adaptation to such conditions requires the modification of gene regulation and metabolic pathways [9,10,11,12,13], thus extremophiles seem to be good potential candidates for novel natural products.

Several reviews discussing natural products from special environments have been published over the last decade, and the topics include natural products from cold water [14,15], polar regions [16,17], and deep sea [18,19]. In 2009 Wilson and Brimble reviewed the studies on the structure of molecules isolated from the extremes of life [6], while those compounds were mainly isolated from bacteria and actinomycetes. Few reviews focused on the secondary metabolites from extremophilic fungi.

This review focuses on the source, chemistry, and biology of novel natural products which were derived from extremophilic fungi. These fungal products are classified according to extremophile classifications, and when a fungal strain falls under multiple classifications, it is grouped under the dominant environmental factor. In addition, the table (Table 1) including the chemical structure types, biological activities and references of all novel natural products will help readers to better understand their underlying potential as drug candidates. It is worth noting that in compiling this review all isolated strains were selected strictly according to the above categories and that strains which do not meet the standards were not cited. For example, one strain was isolated from the deep sea but its underwater depth was less than 3000 m, which did not meet the criteria of piezophiles. Therefore, we did not include it in this review.

## 2. Piezophilic Fungi

**Figure 1:**
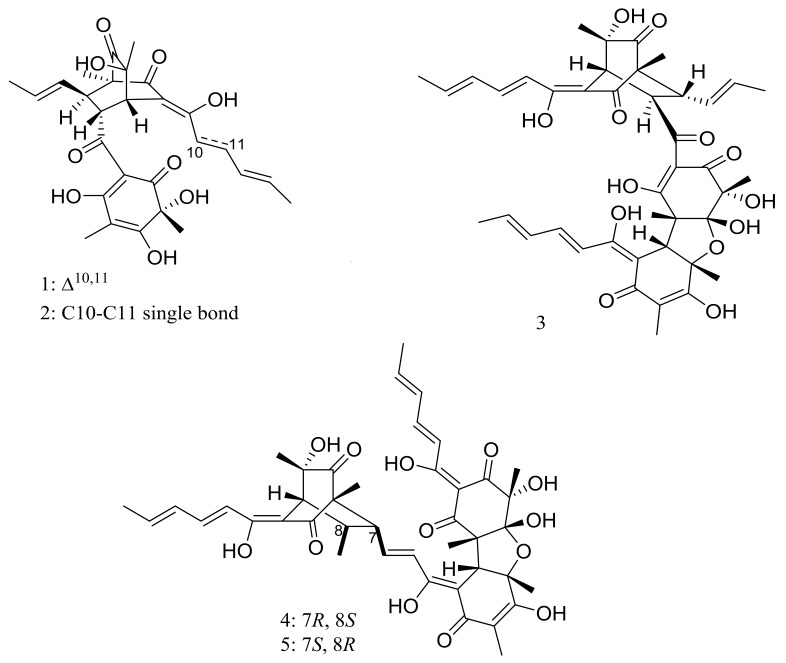

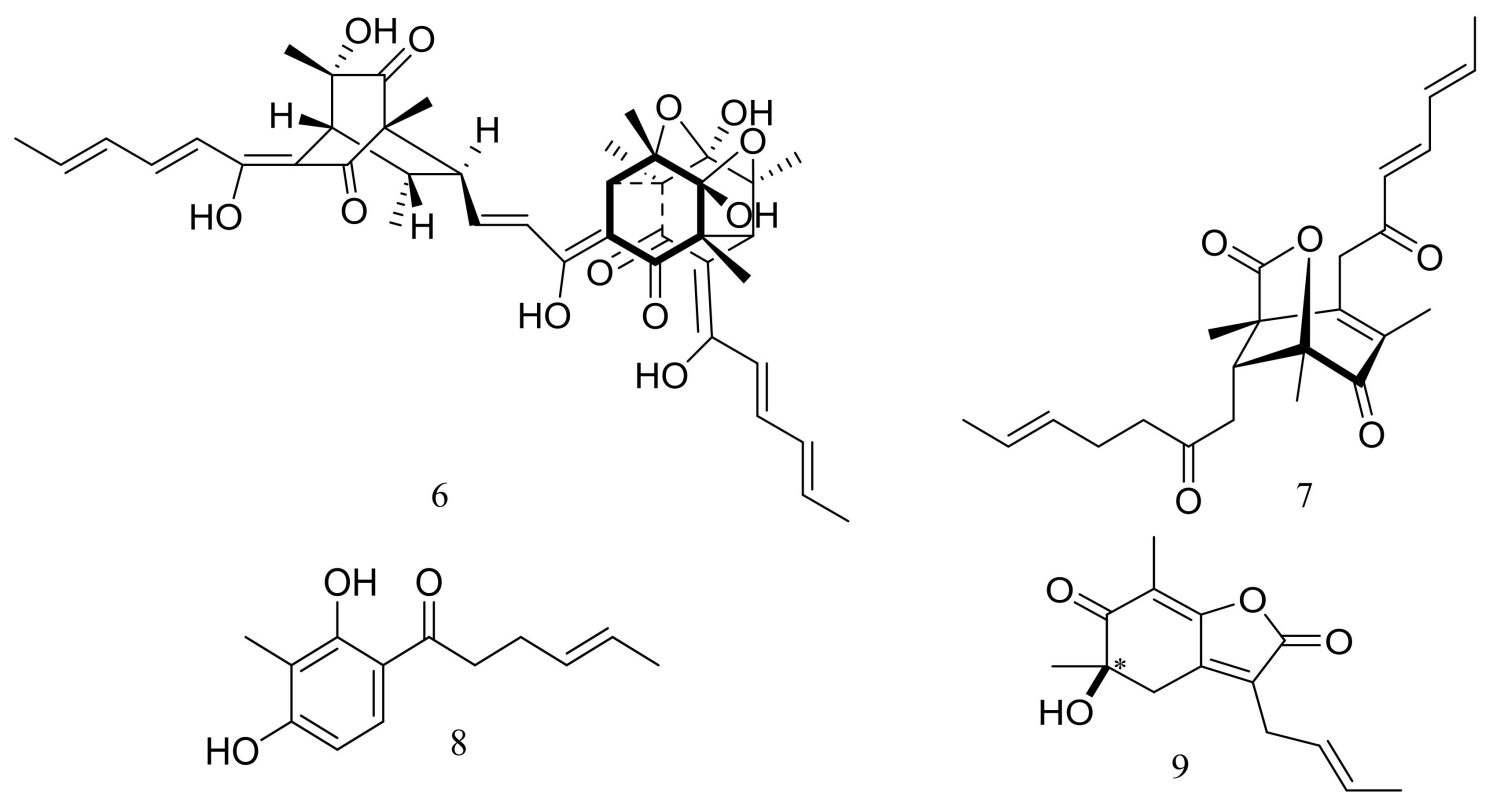
Novel natural products derived from piezophilic fungi (compounds **1**–**9**). * Absolute configuration is not determined.

*Phialocephala* sp. FL30r obtained from an underwater sample (depth 5059 m, the east Pacific) was a powerful producer of diverse sorbicillin-type compounds. Two new bisorbicillinoids, oxosorbiquinol (**1**) and dihydrooxosorbiquinol (**2**) (Figure 1) [20], four new sorbicillin trimers, trisorbicillinones A–D (**3**–**6**) (Figure 1) [21,22], one new sorbicillin dimer, dihydrotrichodermolide (**7**) (Figure 1), one new sorbicillin monomer, dihydrodemethylsorbicillin (**8**) (Figure 1), and one novel benzofuran derivative, phialofurone (**9**) (Figure 1) [23] have been described from this fungal strain since 2007. The cytotoxic activity (IC_50_) of compounds **1** and **2** against several cancer cell lines (P388, HL60, BEL7402, and K562) ranged from 8.9 to 68.2 μM. Compound **3** showed cytotoxic activity against P388 and HL60 cells with IC_50_ values of 9.10 and 3.14 μM respectively, while compounds **4**–**7** exhibited weaker activities against P388 and K562 cells. Compounds **8** and **9** exhibited potent activities against P388 cells with IC_50_ values of 0.1 and 0.2 μM respectively.

**Figure 2:**
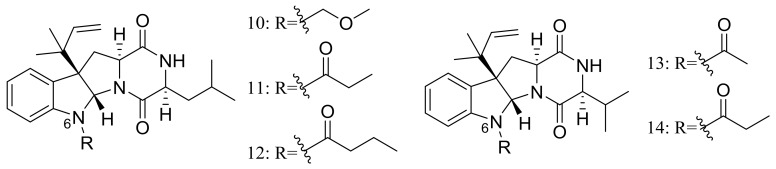
Novel natural products derived from piezophilic fungi (compounds **10**–**14**).

Brevicompanines D–H (**10**–**14**) (Figure 2) are five new diketopiperazine alkaloids produced by the deep-sea sediment-derived fungus *Penicillium* sp. F1 (depth 5080 m). Compounds were assessed for their anti-inflammatory activities on LPS-challenged BV2 cells, **11** and **14** displaying IC_50_ values of 27 and 45 μg/mL respectively, other compounds being found inactive. According to the structure-bioactivity relationship, authors supposed that substitutions at the N-6 position may contribute to the anti-inflammatory activity [24].

**Figure 3:**
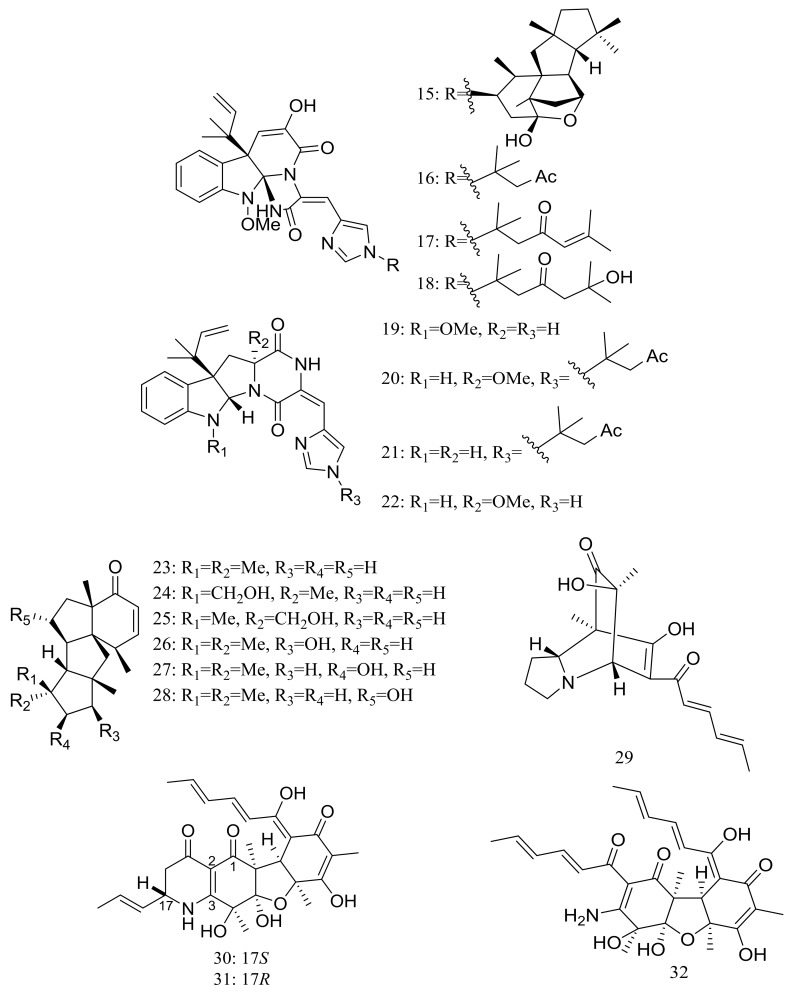

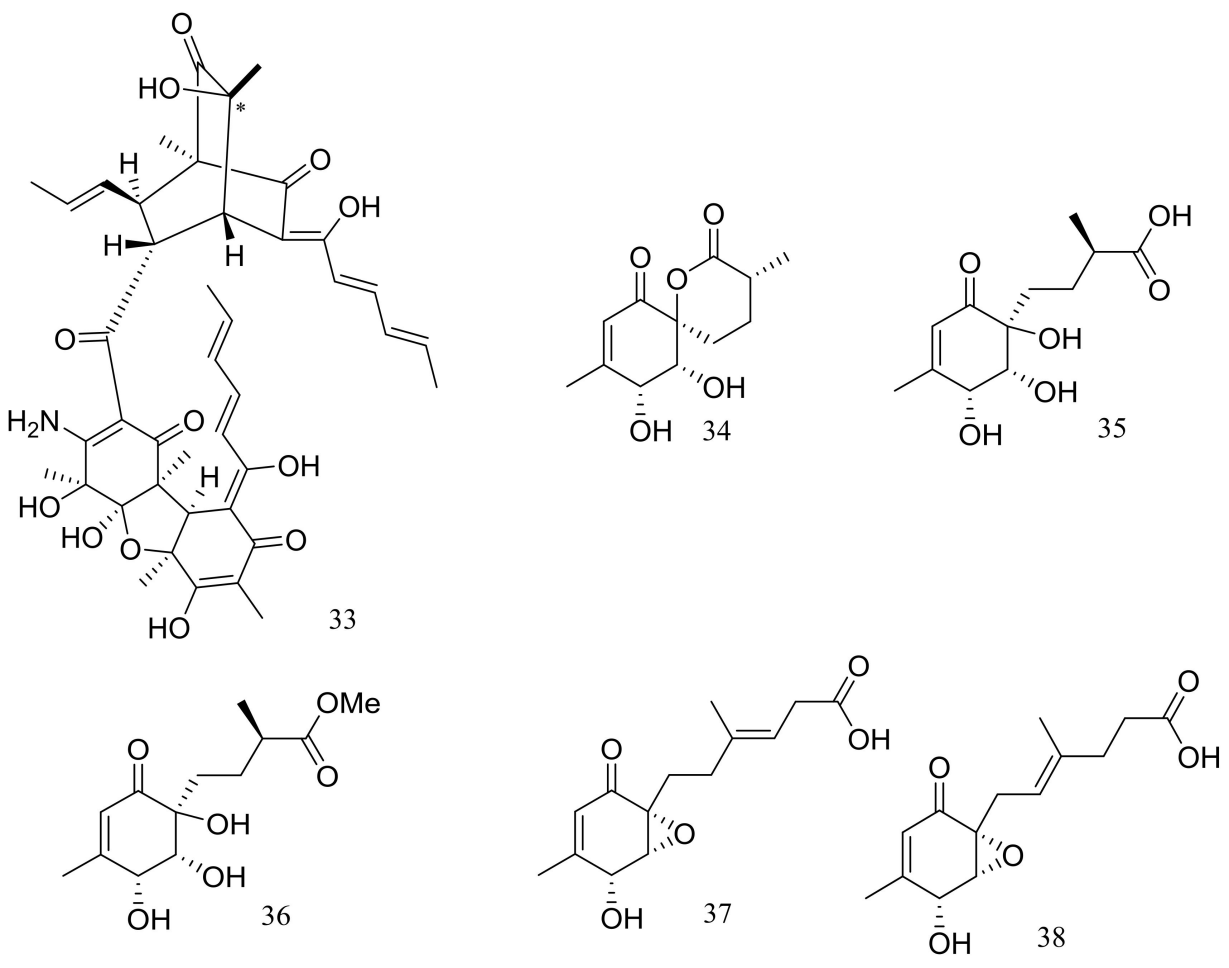
Novel natural products derived from piezophilic fungi (compounds **15**–**38**). * Absolute configuration is not determined.

In 2009 and 2010 several new alkaloids named meleagrins B–E (**15**–**18**) and roquefortines F–I (**19**–**22**) (Figure 3) , along with six new diterpenes named conidiogenones B–G (**23**–**28**) (Figure 3) were described from the deep-sea sediment-derived *Penicillium* sp. F23-2 (depth 5080 m) [25,26]. Compound **15** showed moderate cytotoxic activity against HL60, MOLT4, A549, and BEL7402 cell lines with IC_50_ values ranging from 1.8 to 6.7 μM, while compounds **17** and **18** showed weaker activities against A549 cell line with IC_50_ values of 32.2 and 55.9 μM respectively. Further elucidation of the potential cytotoxic mechanism by flow cytometric analysis indicated that **15** could induce HL60 cells apoptosis at 5 and 10 μM. In addition, compound **24** showed potent selective cytotoxic activity against HL60 and BEL7420 cells with IC_50_ values of 0.038 and 0.97 μM respectively. This study represented the first report on the antitumor activity of the conidiogenone diterpenes. In 2013 five new nitrogen-containing sorbicillinoids, sorbicillamines A–E (**29**–**33**) (Figure 3) were obtained from the PYG liquid culture of this fungal strain. Despite of their interesting structures no cytotoxic activity (HeLa, BEL7402, HEK-293, and HCT116 cell lines) was detected for these metabolites [27]. Guided by the OSMAC approach, in 2015 the same *Penicillium* species afforded another five new ambuic acid analogues named penicyclones A–E (**34**–**38**) (Figure 3) , which exhibited antibacterial activities against *Staphylococcus aureus* with MIC values ranging from 0.3 to 1.0 μg/mL [28].

**Figure 4:**
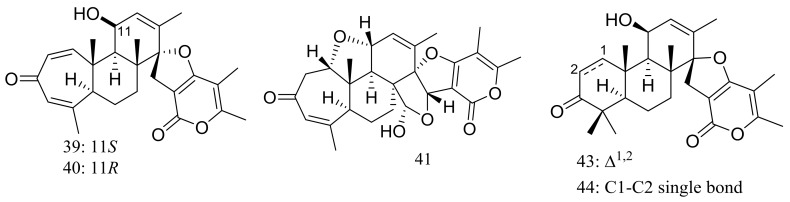

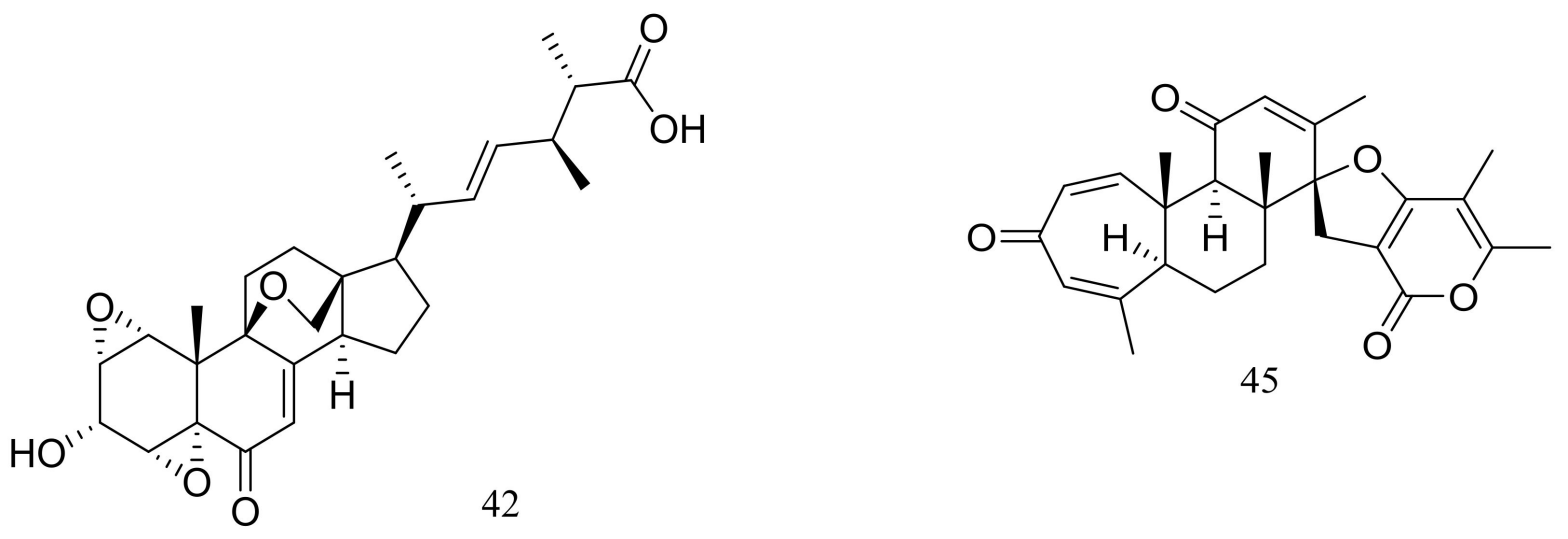
Novel natural products derived from piezophilic fungi (compounds **39**–**45**).

Breviones F–H (**39**–**41**) (Figure 4) were produced by the deep-sea sediment-derived *Penicillium* sp. (MCCC 3A00005) (depth 5115 m, the east Pacific). These three new breviane spiroditerpenoids exhibited cytotoxic activity against HeLa cells with inhibitory rates of 25.2%, 44.9%, and 25.3% at 10 μg/mL, respectively. Effects on HIV-1 inhibition in C8166 cells were tested and an EC_50_ value for compound **39** was 14.7 μM [29]. From the same *Penicillium* strain one new polyoxygenated sterol named sterolic acid (**42**) and three new breviane spiroditerpenoids namend breviones I–K (**43**–**45**) (Figure 4) were published later. Compound **43** exhibited cytotoxic activity against MCF7 and A549 cells with IC_50_ values of 7.44 and 32.5 μM respectively [30].

**Figure 5:**
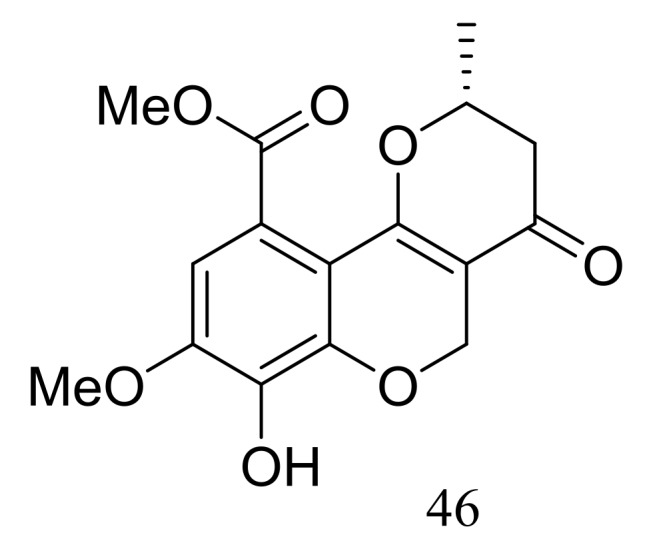
Novel natural products derived from piezophilic fungi (compound **46**).

Ascomycotin A (**46**) (Figure 5) was isolated from the deep-sea sediment-derived *Ascomycota* sp. Ind19F07 (depth 3614 m, the Indian Ocean) grown on the rice solid media. No antimicrobial activity (*Acinetobacter baumannii* (ATCC 19606), *Klebsiella pneumoniae* (ATCC 13883), *Escherichia coli* (ATCC 25922), *Staphyloccocus aureus* (ATCC 29213) and *Enterococcus faecalis* (ATCC 29212)) was detected [31].

**Figure 6:**
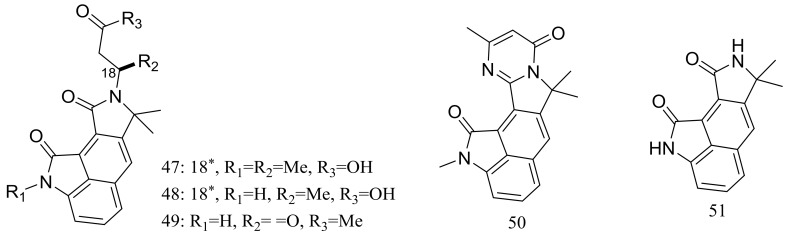

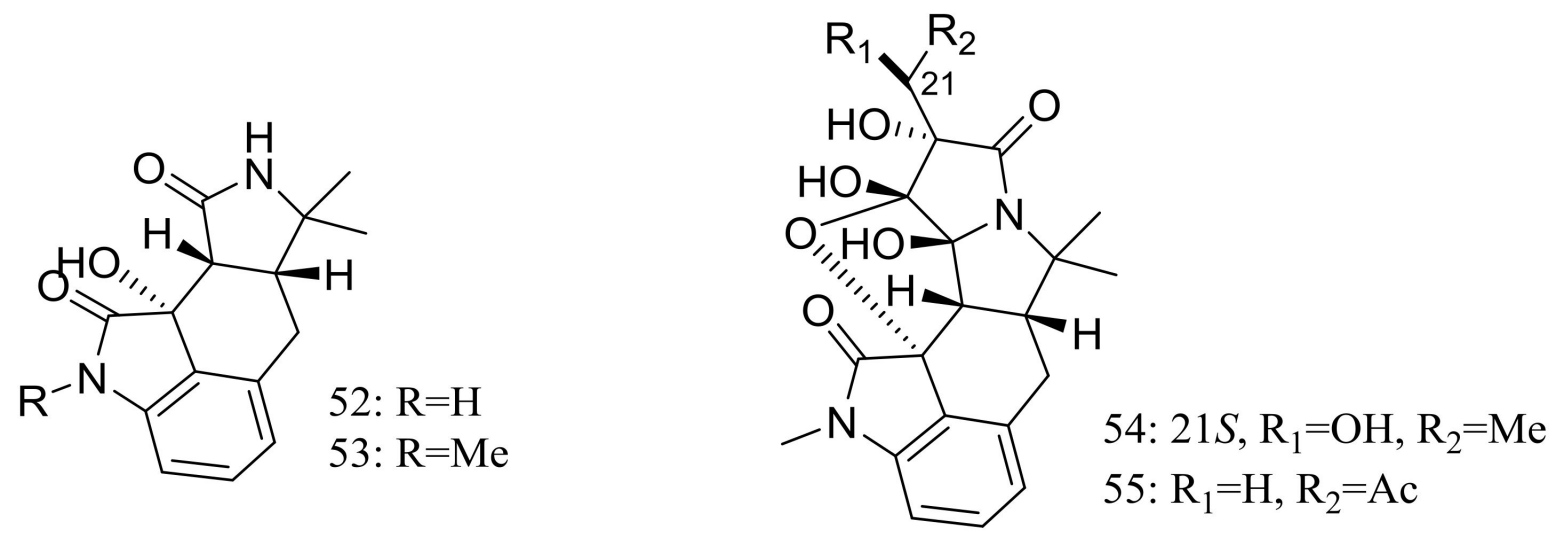
Novel natural products derived from piezophilic fungi (compounds **47**–**55**). * Absolute configuration is not determined.

Cyclopiamides B–J (**47**–**55**) (Figure 6), nine new cyclopiamide analogues belonging to oxindole alkaloids were produced by the deep-sea-derived fungal strain *Penicillium commune* DFFSCS026 (depth 3563 m, the South China Sea). Toxic activities against brine shrimp of all nine compounds were almost the same with IC_50_ values ranging from 14.1 to 38.5 μg/mL, which suggested that structural modifications at the indole system might not significantly affect their toxic activities. No cytotoxic (HepG-2 and HeLa cell lines) or antiviral (N1H1) activities were detected [32].

**Figure 7:**
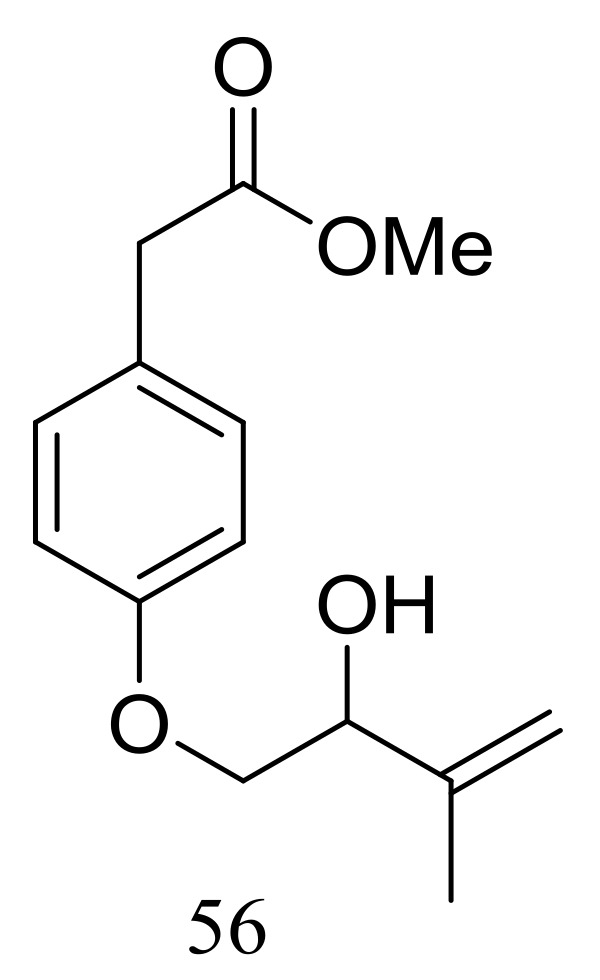
Novel natural products derived from piezophilic fungi (compound **56**).

A new hydroxyphenylacetic acid named westerdijkin A (**56**) (Figure 7) was isolated from a deep-sea sediment-derived fungal strain *Aspergillus westerdijkiae* SCSIO 05233 (depth 4593 m, the South China Sea). Neither antimicrobial (*Escherichia coli*, *Bacillus subtilis*, *Bacillus pumilus*, *Staphylococcus aureus*, and *Candida albicans*), anticancer (K562 and HL-60 cell lines), nor antifouling (*Balanus amphitrite*) activities were detected [33].

**Figure 8:**
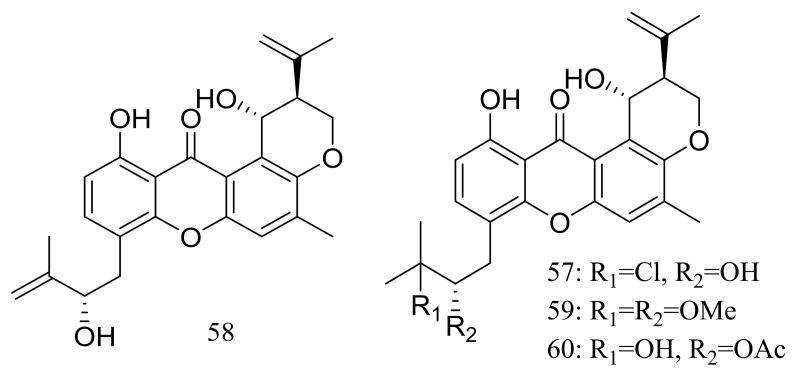
Novel natural products derived from piezophilic fungi (compounds **57**–**60**).

Four new prenylxanthones, emerixanthones A–D (**57**–**60**) (Figure 8) were isolated from *Emericella* sp. SCSIO 05240 (depth 3258 m, the South China Sea). Compounds **57** and **59** exhibited weak antibacterial activities against six pathogens (*Escherichia coli* (ATCC 29922), *Klebsiella pneumoniae* (ATCC 13883), *Staphylococcus aureus* (ATCC 29213), *Enterococcus faecalis* (ATCC 29212), *Acinetobacter baumannii* (ATCC 19606), and *Aeromonas hydrophila* (ATCC 7966), while **60** displayed mild antifungal activity against six agricultural pathogens (*Fusarium* sp., *Penicillium* sp., *Aspergillus niger*, *Rhizoctonia solani*, *Fusarium oxysporum f.* sp. *niveum*, and *Fusarium oxysporum f.* sp. *cucumeris*). The biosynthetic pathway of these metabolites was proposed [34].

**Figure 9:**
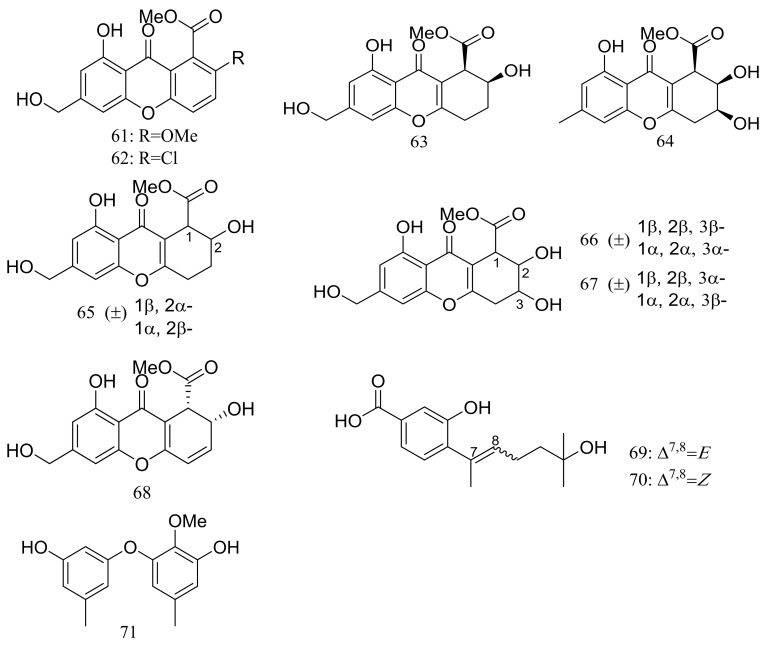
Novel natural products derived from piezophilic fungi (compounds **61**–**71**).

Engyodontiumones A–J (**61**–**70**) and 2-methoxyl cordyol C (**71**) (Figure 9) have been described as metabolites of *Engyodontium album* DFFSCS021 taken from a 3739 m deep-sea sediment sample in the South China Sea. Compound **68** exhibited cytotoxic activity against U937 cells (IC_50_ 4.9 μM) and antimicrobial activity against *Escherichia coli* and *Bacillus subtilis* at a concentration of 25 μg/disc [35].

**Figure 10:**
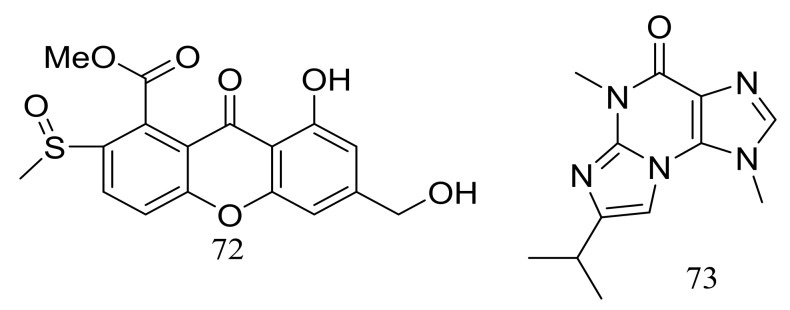
Novel natural products derived from piezophilic fungi (compounds **72**–**73**).

The solid fermentation of the deep-sea fungus *Aspergillus* sp. SCSIO Ind09F01 (depth 4530 m, the Indian Ocean) yielded a new xanthone named sydoxanthone C (**72**) and a new alkaloid named acremolin B (**73**) (Figure 10). Two compounds exhibited no cytotoxic (Hela, DU145, and U937 cell lines) or COX-2 inhibitory activities [36].

**Figure 11:**
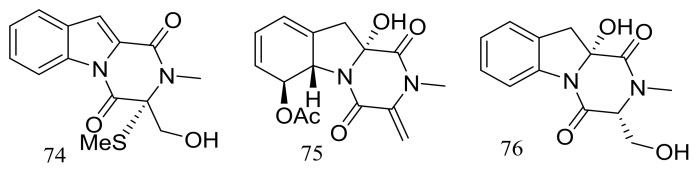
Novel natural products derived from piezophilic fungi (compounds **74**–**76**).

Dichotocejpins A–C (**74**–**76**) (Figure 11) are three new diketopiperazines produced by *Dichotomomyces cejpii* FS110 (depth 3941 m). The inhibitory activity of compound **74** (IC_50_ 138 μM) against α-glucosidase was much stronger than that of the positive control acarbose (IC_50_ 463 μM) [37].

**Figure 12:**
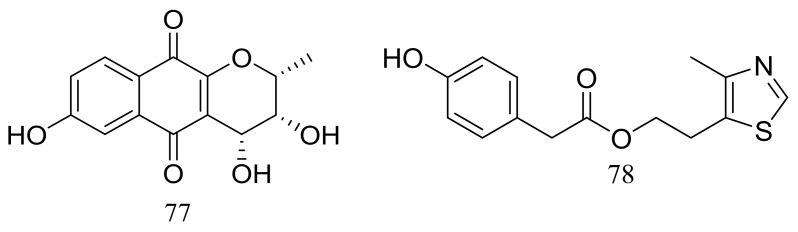
Novel natural products derived from piezophilic fungi (compounds **77**–**78**).

Acaromycin A (**77**) (Figure 12), a new naphtha-[2,3-b] pyrandione analogue and acaromyester A (**78**) (Figure 12), a new thiazole analogue were isolated from *Acaromyces ingoldii* FS121 (depth 3415 m, the South China Sea). Pronounced cytotoxic activities against four cancer cell lines (MCF-7, NCI-H460, SF-268, and HepG-2) were described for compound **77** with IC_50_ values less than 10 μM [38].

**Figure 13:**
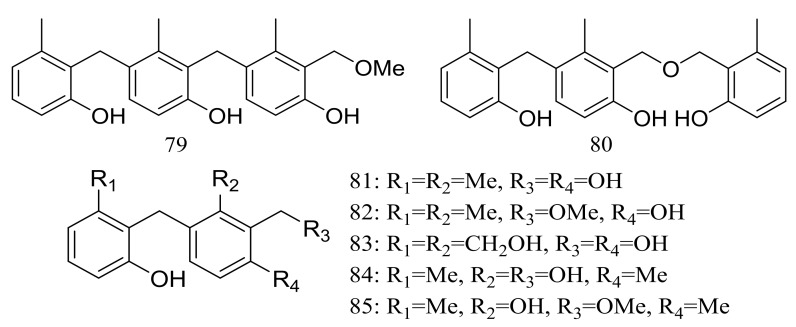
Novel natural products derived from piezophilic fungi (compounds **79**–**85**).

The trimeric peniphenylanes A–B (**79**–**80**) and dimeric peniphenylanes C–G (**81**–**85**) (Figure 13) are seven new 6-methylsaligenin derivatives obtained from *Penicillium fellutanum* HDN14-323 (depth 5752 m, the Indian Ocean). When tested for cytotoxic activity compound **82** proved to be the best active to HeLa cells (IC_50_ 9.3 μM) [39].

**Figure 14:**
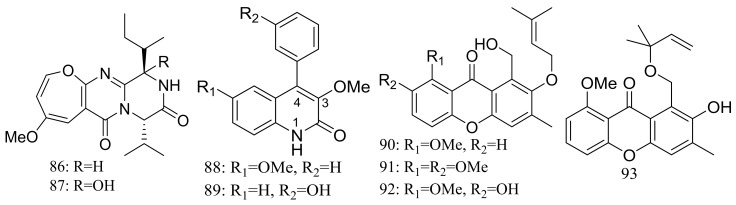
Novel natural products derived from piezophilic fungi (compounds **86**–**93**).

The deep-sea sediment-derived fungus *Aspergillus versicolor* SCSIO 05879 (depth 3972 m, the Indian Ocean) was found to produce two new oxepine-containing diketopiperazine-type alkaloids named versicoloids A–B (**86**–**87**) (Figure 14), two new 4-aryl-quinolin-2-one alkaloids (**88**–**89**) (Figure 14), and four new prenylated xanthones named versicones A–D (**90**–**93**) (Figure 14). Compounds **86** and **87** displayed the same MIC valued of 1.6 μg/mL against *Colletotrichum acutatum* [40].

**Figure 15:**
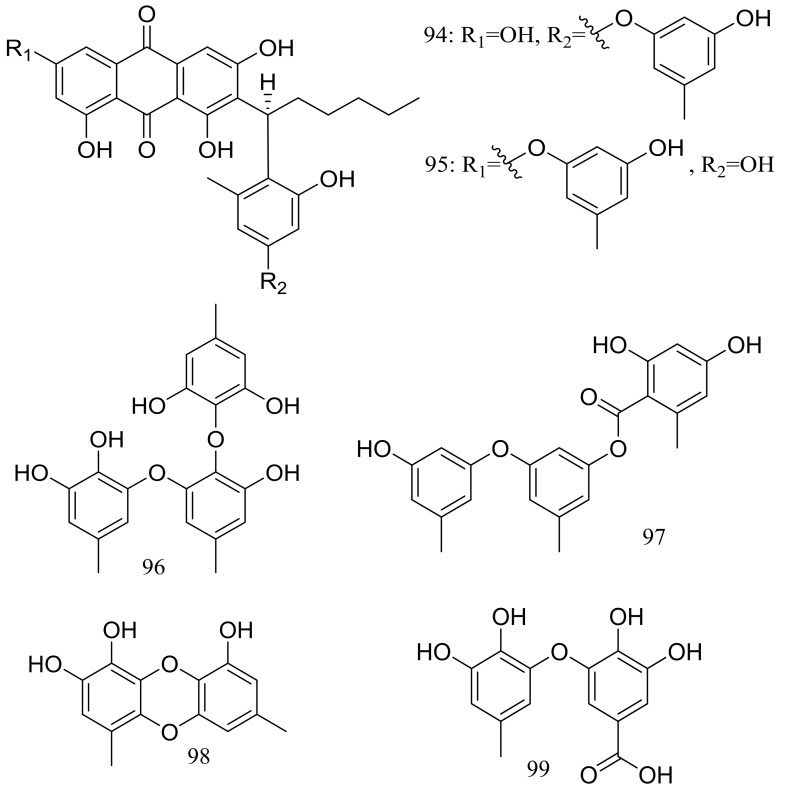
Novel natural products derived from piezophilic fungi (compounds **94**–**99**).

Aspergilols A–F (**94**–**99**) (Figure 15) were isolated from fermentations of the deep-sea fungus *Aspergillus versicolor* (A-21-2-7) (depth 3002 m, the South China Sea). Compound **98** significantly activated the Nrf2, which regulated the expression of antioxidant proteins that protect against oxidant damage [41].

**Figure 16:**
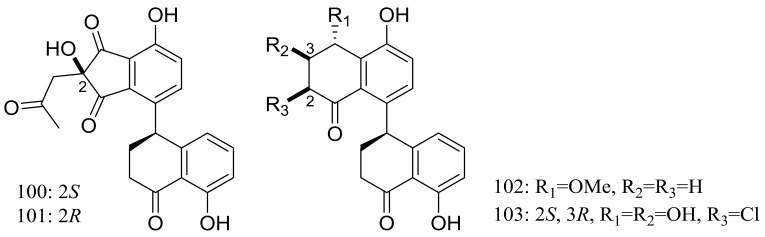
Novel natural products derived from piezophilic fungi (compounds **100**–**103**).

The clindanones A–B (**100**–**101**) and cladosporols F–G (**102**–**103**) (Figure 16) are four new polyketides isolated from the deep-sea fungus *Cladosporium cladosporioides* HDN14-342 (depth 3471 m, the Indian Ocean). Compounds **102**–**103** showed moderate cytotoxic activity against HeLa, K562, and HCT-116 cell lines with IC_50_ values of 3.9 to 23.0 μM [42].

**Figure 17:**
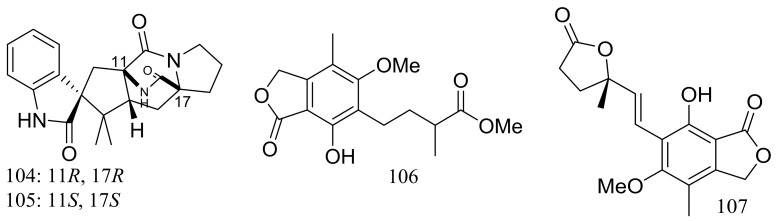
Novel natural products derived from piezophilic fungi (compounds **104**–**107**).

The fungal strain *Penicillium brevicompactum* DFFSCS025 (depth 3928 m, the South China Sea) produced two new brevianamides, brevianamids X–Y (**104**–**105**) and two new mycochromenic acid derivatives (**106**–**107**) (Figure 17). Compound **106** showed strong antilarval activity against *Bugula neritina* with an EC_50_ value of 13.7 μM [43].

**Figure 18:**
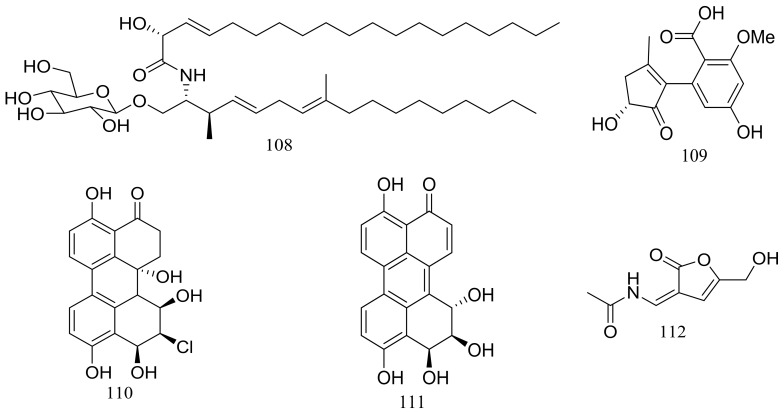
Novel natural products derived from piezophilic fungi (compounds **108**–**112**).

In exploring for new BRD4 inhibitors, five new compounds including one new cerebroside (**108**) (Figure 18), one new alternaric acid (**109**) (Figure 18), two new perylenequinones (**110**–**111**) (Figure 18), and 2-(*N*-vinylacetamide)-4-hydroxymethyl-3-ene-butyrolactone (**112**) (Figure 18) were isolated from fermentations of *Alternaria* sp. NH-F6 (depth 3927 m, the South China Sea). Compound **111** was a potent inhibitor with an inhibition rate of 88.1% at 10 μM, while **110** had a moderate inhibition at rate of 57.7% at the same concentration [44].

**Figure 19:**
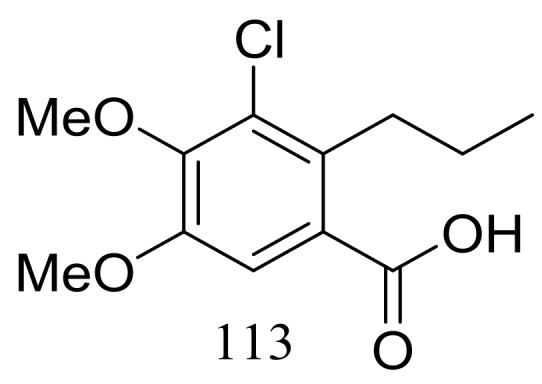
Novel natural products derived from piezophilic fungi (compound **113**).

Engyodontiumin A (**113**) (Figure 19) was produced by the deep-sea-derived fungus *Engyodontium album* (depth 3542 m, the Atlantic Ocean). This novel benzoic acid derivative displayed moderate antibacterial activity against *Aspergillus niger*, MRSA, *Vibrio vulnificus*, *Vibrio rotiferianus*, and *Vibrio campbellii*. The experimental data on the antimicrobial activity were not provided in the original article [45].

**Figure 20:**
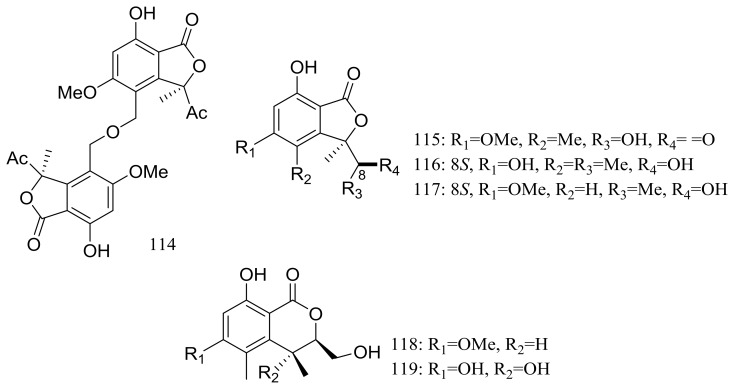
Novel natural products derived from piezophilic fungi (compounds **114**–**119**).

In exploration for novel bioactive marine natural products, four new isobenzofuanones named leptosphaerins JM (**114**–**117**) and two new isochromenones named clearanols I–J (**118**–**119**) (Figure 20) were isolated from *Leptosphaeria* sp. SCSIO 41005 (depth 3614 m, the Indian Ocean). When evaluated for biological activity, no cytotoxicity (K562, MCF-7, and SGC7901 cell lines) or antiviral activity (H3N2, EV71, and HIV viruses) was detected [46].

**Figure 21:**
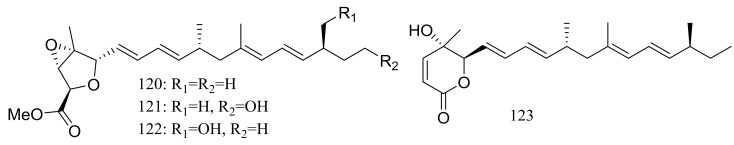
Novel natural products derived from piezophilic fungi (compounds **120**–**123**).

A mixed culture of the deep-sea-derived fungus *Talaromyces aculeatus* (depth 3386 m, the Indian Ocean) and the mangrove-derived fungus *Penicillium variabile* (Fujian Province of China) afforded four new polyketides, penitalarins A–C (**120**–**122**) and nafuredin B (**123**) (Figure 21). None of these compounds was produced by either of the two fungi when cultured alone under the same conditions. Compound **123** inhibited a panel of cancer cell lines (HeLa, K562, HCT-116, HL-60, A549, and MCF-7) with IC_50_ values ranging from 1.2 to 9.8 μM (doxorubicin as positive control IC_50_ 0.2 to 0.8 μM) [47].

**Figure 22:**
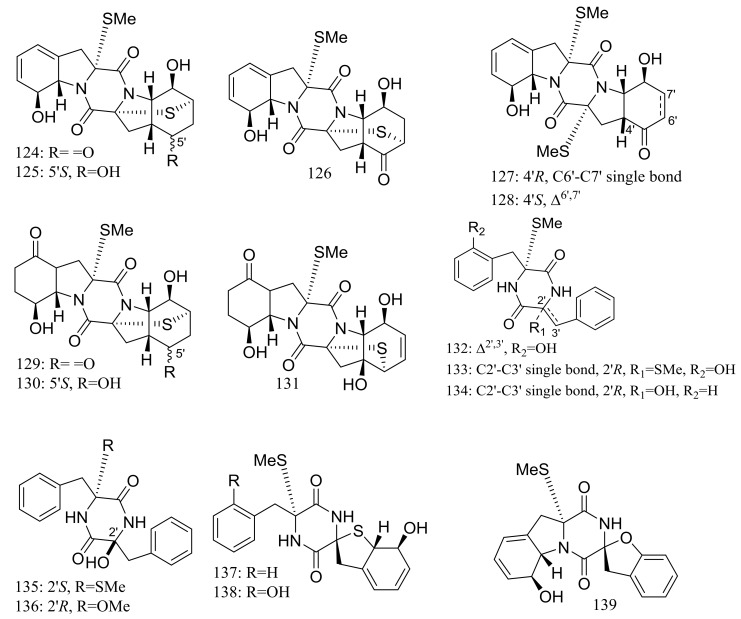

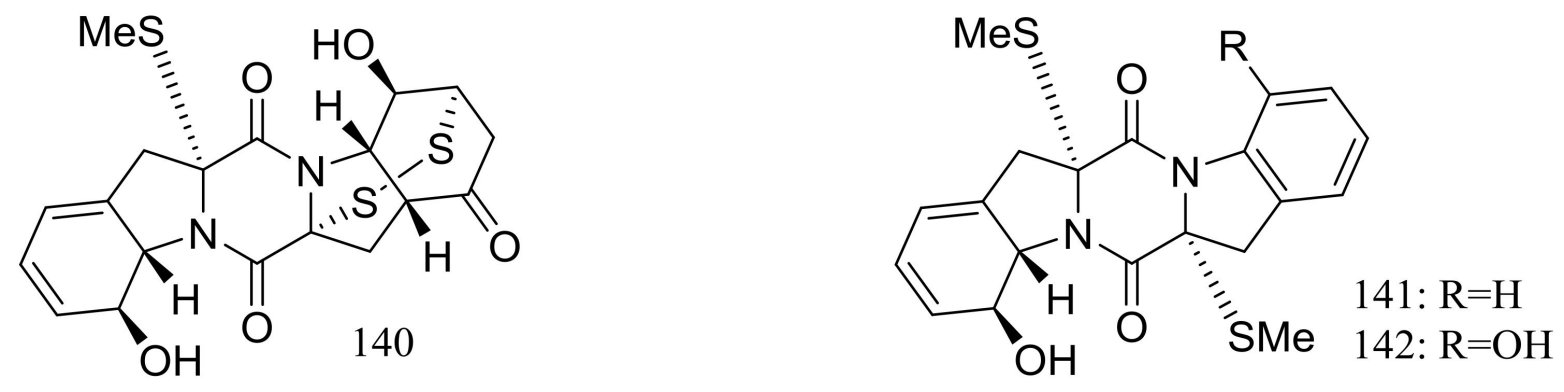
Novel natural products derived from piezophilic fungi (compounds **124**–**142**).

Nineteen new thiodiketopiperazine-type alkaloids named eutypellazines A–S (**124**–**142**) (Figure 22) [48,49] were produced by the marine-derived fungus *Eutypella* sp. MCCC 3A00281 (depth 5610 m, the South Atlantic Ocean). Inhibitory effects on HIV-1 replication in pNL4.3Env-.Luc co-transfected 293T cells were tested and IC_50_ values for compounds **124**–**135** ranged from 3.2 to 18.2 μM (EFV as the positive control IC_50_ 0.1 μM). In addition, compound **133** could reactivate latent HIV-1 in J-Lat A2 cells in a dose-dependent manner. When tested for antimicrobial activity compounds **139**–**142** were active to *Staphylococcus* aureus ATCC 25923 and vancomycin-resistant enterococci with MIC values of 32/32, 16/16, 32/32, and 16/32 μM respectively.

**Figure 23:**
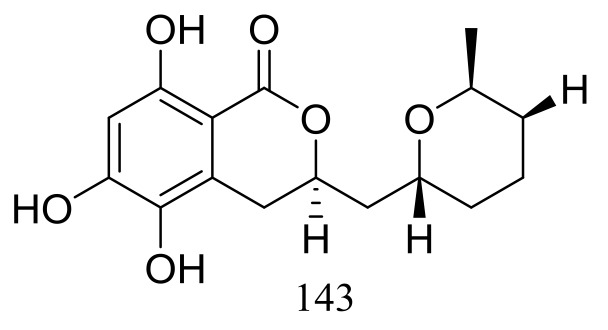
Novel natural products derived from piezophilic fungi (compound **143**).

The cultured broth of the sea cucumber-derived fungus *Penicillium coralligerum* YK-247 (depth 3064 m, São Paulo Plateau, off Brazil) potently inhibited the growth of *Saprolegnia parasitica*. Further chromatographic fractionation of the cultured broth led to the isolation of cladomarine (**143**) (Figure 23) which showed selective antimicrobial activity against *Saprolegnia parasitica* and *Pythium* sp. sakari1 at a concentration of 10 μg/disc [50].

## 3. Psychrophilic Fungi

**Figure 24:**
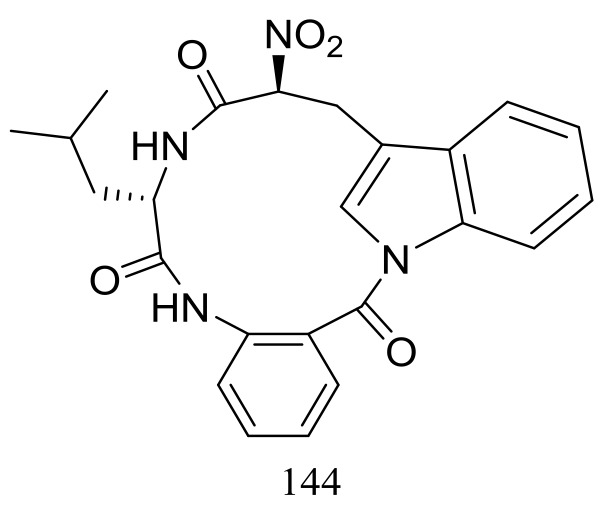
Novel natural products derived from psychrophilic fungi (compound **144**).

Psychrophilin D (**144**) (Figure 24), a new cyclic nitropeptide was isolated from *Penicillium algidum* derived from a soil sample in Greenland. This compound exhibited moderate cytotoxic activity against P388 murine leukaemia cells with an ID_50_ value of 10.1 μg/mL. When evaluated for antimicrobial, antiviral, anticancer and antiplasmodial activities compound **144** proved to be inactive [51].

**Figure 25:**
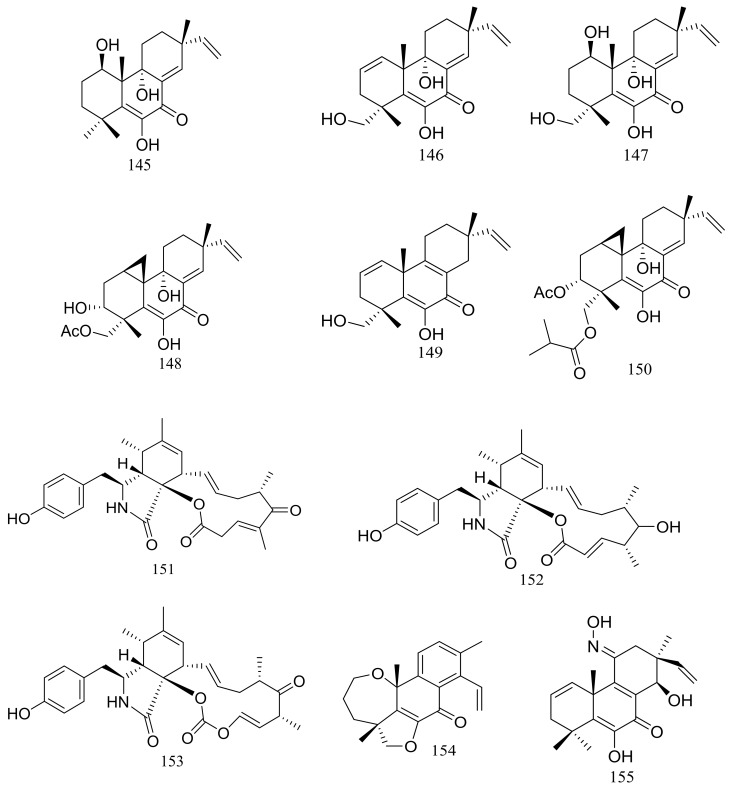

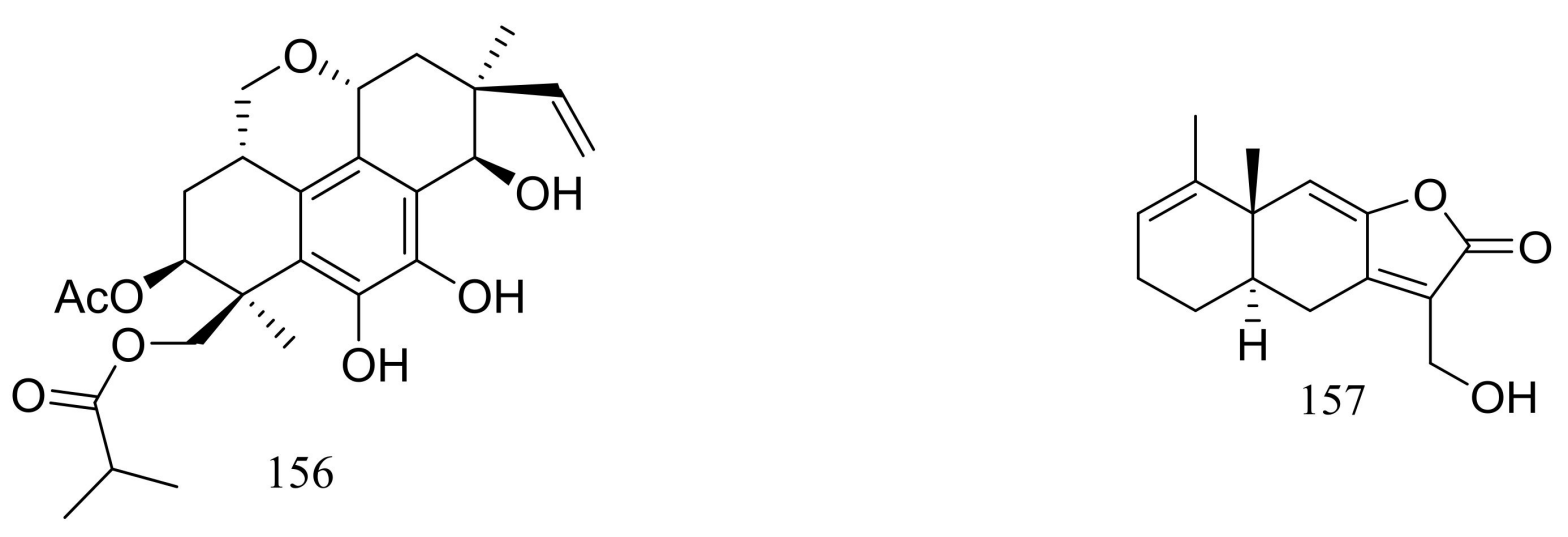
Novel natural products derived from psychrophilic fungi (compounds **145**–**157**).

In 2005 Oh et al. discovered libertellenones A–D (**145**–**148**) (Figure 25) when co-cultured a marine-derived fungus with a unicellular marine bacterium. Libertellenone D (**148**) demonstrated potent cytotoxicity (IC_50_ 0.76 μM) against HCT-116 cell line, whereas the other libertellenones exhibited weaker activities (IC_50_ 15, 15 and 53 μM respectively) [52]. In 2014 libertellenones G (**149**) and H (**150**) (Figure 25) together with **145** and **147** were isolated from *Eutypella* sp. D-1, which was derived from a soil sample collected on London Island of Kongsfjorden of Ny-Ålesund District (altitude of 100 m), Arctic. Compound **149** showed moderate antibacterial activity against *Escherichia coli*, *Bacillus subtilis*, and *Staphylococcus aureus*. Compound **150** showed slight cytotoxicity against several cancer cell lines (MCF-7, H460, U251, SW-1990, Hela, Huh-7, and SG7901) with IC_50_ values between 3.31 and 44.1 μM. According to the structure-bioactivity relations, the cyclopropane ring in **148** and **150** appears to be an important structural feature associated with their biological activity [53]. Later Chu’s group found that **150** biosynthesis was significantly elevated (16.4 folds) with ethanol treatment, and further study showed that the gene transcription levels of 3-hydroxy-3-methyl glutaric acyl coenzyme A reductase and geranylgeranyl diphosphate synthase were up-regulated by ethanol stimulation [54]. Several new compounds including cytochalasins Z_24_, Z_25_, Z_26_ (**151**–**153**) (Figure 25) [55], eutypenoids A–C (**154**–**156**) (Figure 25) [56], and *eut*-Guaiane sesquiterpene (**157**) (Figure 25) [57] have been described from the same fungal strain since 2014. Compound **151** exhibited a moderate cytotoxicity against MCF-7 cells with an IC_50_ value of 9.33 μM. Compound **155** was able to suppress the proliferation of BALB/c mice splenocytes under ConA induction. Antibacterial activity (*Escherichia coli*, *Bacillus subtilis*, and *Staphylococcus aureus*) of compound **157** was comparable to that of ampicillin but cytotoxic activity against SGC7901 cells was very weak (IC_50_ 39.8 μM).

**Figure 26:**
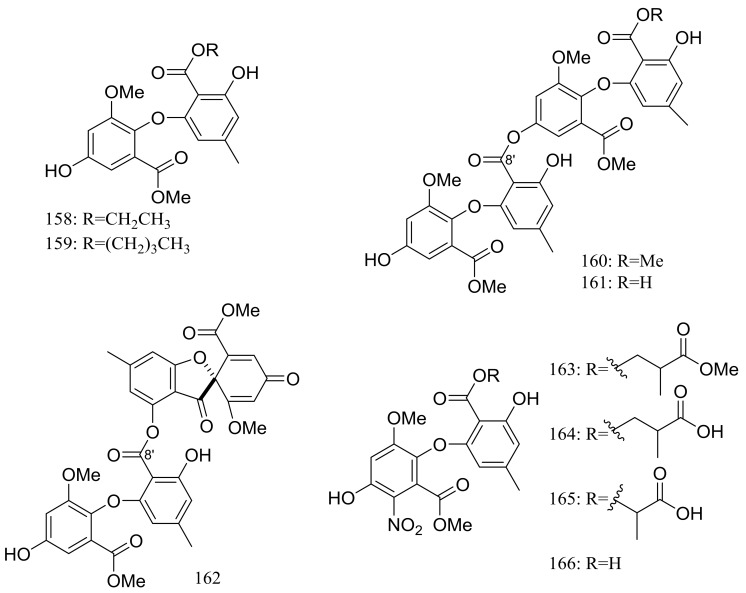
Novel natural products derived from psychrophilic fungi (compounds **158**–**166**).

In search for new antifungal and antibacterial natural products, five asterric acid derivatives named ethyl asterrate (**158**), n-butyl asterrate (**159**) and geomycins A–C (**160**–**162**) (Figure 26) were isolated from an Antarctic *Geomyces* species. Compound **161** showed significant antifungal activity against *A. fumigatus* (ATCC 10894) with IC_50_/MIC values of 0.86/29.5 µM (the positive control fluconazole IC_50_/MIC 7.35/163.4 µM). Compound **162** exhibited moderate antimicrobial activity against both Gram-positive and Gram-negative bacteria with IC_50_ values ranging from 12.9 to 36.2 µM [58]. In 2015 four nitroasterric acid derivatives named pseudogymnoascins A–C (**163**–**165**) and 3-nitroasterric acid (**166**) (Figure 26) were described as metabolites of a sponge-associated fungus *Pseudogymnoascus* sp. F09-T18-1, which was collected from the King George Island of Antarctic. No antimicrobial activity was observed at MIC > 64 μg/mL. Compared with compounds **161** and **162**, the lack of antimicrobial activities of compounds **163**–**166** suggested the activity lied in the size of substituent at C-8′ and/or the presence of the nitro group in the molecule [59].

**Figure 27:**
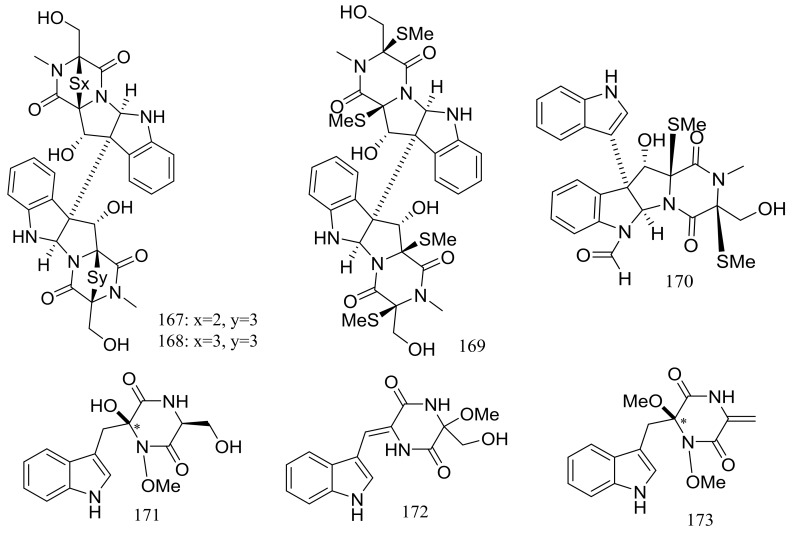
Novel natural products derived from psychrophilic fungi (compounds **167**–**173**). * Absolute configuration is not determined.

Several piperazine-type compounds, chetracins B–D (**167**–**169**) and oidioperazines A–D (**170**–**173**) (Figure 27) were produced by the soil-derived Antarctic fungus *Oidiodendron truncatum* GW3-13 which was obtained near the Great Wall station (Chinese Antarctic station). When tested for cytotoxic activities against a panel of cancer cell lines (HCT-8, Bel-7402, BGC-823, A549, and A2780) compound **167** proved to be the most active (IC_50_ 0.003 to 0.028 μM), whereas **168** and **169** were less active (IC_50_ 0.14 to 1.83 μM) [60].

**Figure 28:**
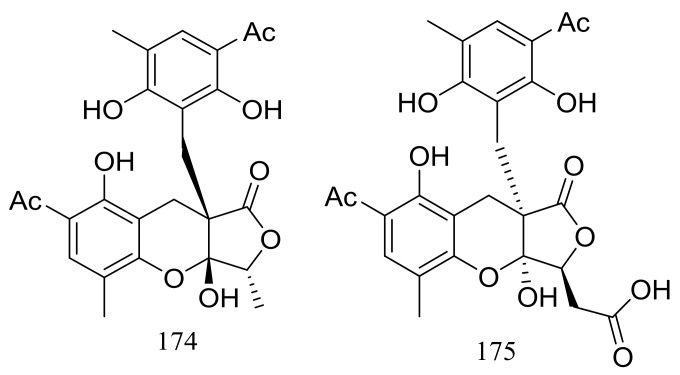
Novel natural products derived from psychrophilic fungi (compounds **174**–**175**).

Two highly oxygenated polyketides, penilactones A and B (**174** and **175**) (Figure 28) featuring a new carbon skeleton formed from two 3,5-dimethyl-2,4-diol-acetophenone units and a γ-butyrolactone moiety were produced by the Antarctic marine-derived fungus *Penicillium crustosum* PRB-2. A plausible biogenetic pathway was proposed in the original article. Effects on NF-κB inhibition (in transient transfection and reporter gene expression assay) in RAW264.7 cells were tested and compound **174** showed very weak activity with a rate of 40% at 10 mM (the positive control PDTC inhibitory rate 85% at 0.1 mM) [61].

**Figure 29:**
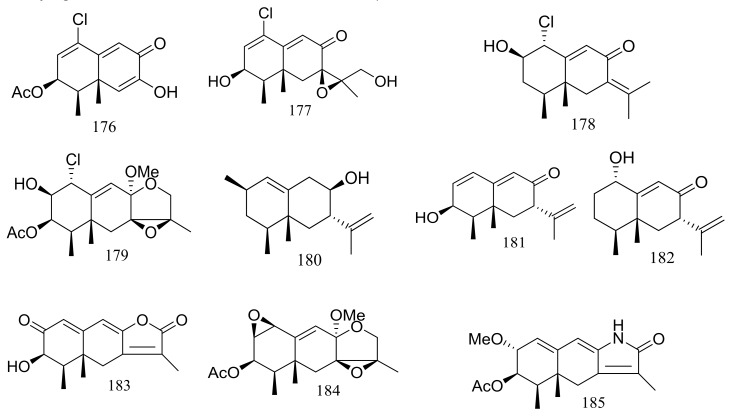
Novel natural products derived from psychrophilic fungi (compounds **176**–**185**).

Several interesting eremophilane-type sesquiterpenes with high structural diversity have been described for *Penicillium* sp. PR19N-1 derived from a sludge sample in Prydz Bay (−1000 m), Antarctica. In 2013 four new chloro-eremophilane sesquiterpenes (**176**–**179**) (Figure 29) were isolated from this fungal strain and the plausible metabolic network was proposed. Compound **176** displayed modest cytotoxic activity against HL-60 and A549 cell lines with IC_50_ values of 11.8 and 12.2 μM respectively, whereas the other compounds exhibited no activities. [62]. Soon later another five new eremophilane-type sesquiterpenes (**180**–**184**) and a rare lactam-type eremophilane (**185**) (Figure 29) were isolated from the same *Penicillium* strain. When tested for cytotoxic activities against HL-60 and A-549 cells only **180** and **184** proved to be active and compound **184** displayed strong cytotoxic activity against A-549 cells with an IC_50_ value of 5.2 μM [63].

**Figure 30:**
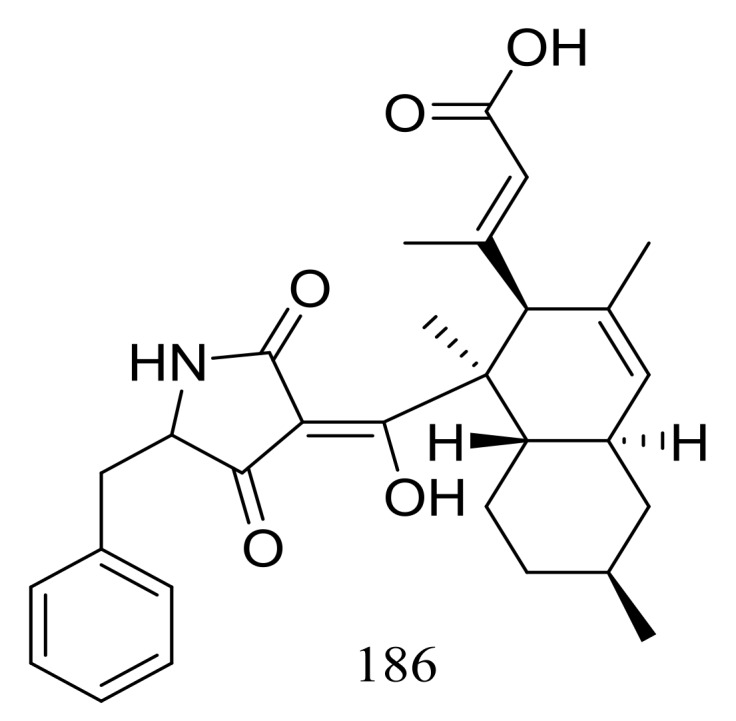
Novel natural products derived from psychrophilic fungi (compound **186**).

Two different Lindgomycetaceae strains KF970 and LF327 obtained from different marine habitats (Antarctic and the Kiel Fjord, Baltic Sea) both produced lindgomycin (**186**) (Figure 30), an unusual polyketide with a unique 5-benzylpyrrolidine-2,4-dione unit at the tetramic acid substructure. Antibiotic activity (*Bacillus subtilis*, *Staphylococcus aureus*, and methicillin-resistant *Staphylococcus aureus*) of compound **186** were two times less than that of chloramphenicol (the positive control) [64].

**Figure 31:**
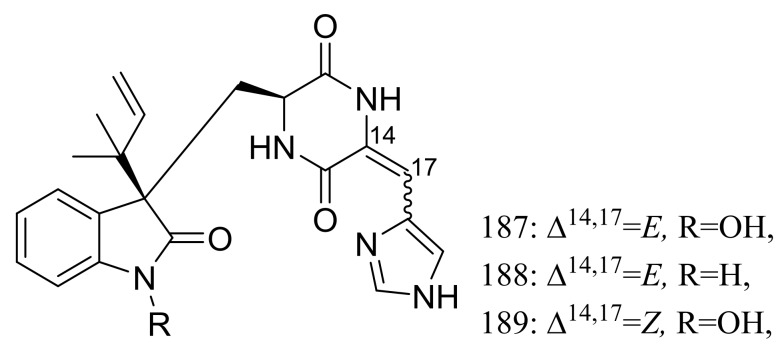
Novel natural products derived from psychrophilic fungi (compounds **187**–**189**).

The psychrotolerant fungus *Penicillium* sp. SCSIO 05705 collected nearby the Great Wall station (Chinese Antarctic station) afforded three new indolyl diketopiperazine derivatives, penillines A–B (**187**–**188**) and isopenilline A (**189**) (Figure 31). In the general bioactivity profiling programs including antiviral, cytotoxic, antibacterial and antituberculosis evaluation, all compounds were found inactive [65].

**Figure 32:**
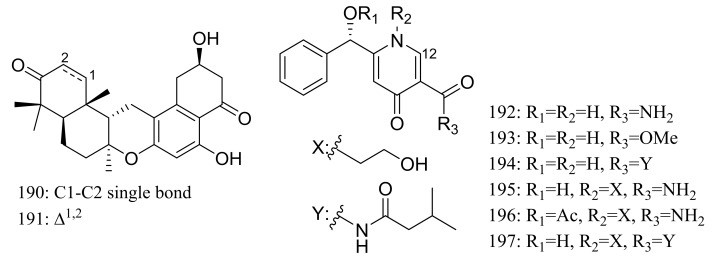
Novel natural products derived from psychrophilic fungi (compounds **190**–**197**).

Chrodrimanins I (**190**) and J (**191**) (Figure 32), two new meroterpenoids were isolated from the moss-derived *Penicillium funiculosum* GWT2-24, collected at the China Great Wall Station in Antarctica. Distinguished from the reported chrodrimanins, compounds **190** and **191** possessed a unique cyclohexanone instead of a δ-lactone ring. Neither antiviral activity (H1N1) nor cytotoxic activity (K562, HL60, HeLa, and A549 cell lines) was detected [66]. From the same fungal strain, six new pyridine alkaloids named penipyridones A–F (**192**–**197**) (Figure 32) were published later. When screened for lowering of oleic acid elicited lipid accumulation in HepG2 hepatocytes, compounds **192**, **193** and **196** remarkably reduced intracellular lipid accumulation as well as the total cholesterol and triglyceride quantification at 10 μM [67].

**Figure 33:**
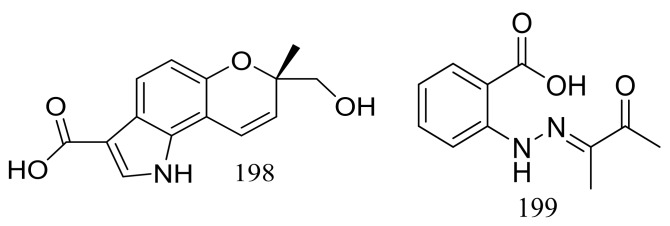
Novel natural products derived from psychrophilic fungi (compounds **198**–**199**).

Exopisiod B (**198**) and farylhydrazone C (**199**) (Figure 33) were produced by a soil-derived fungus *Penicillium* sp. HDN14-431 collected from mesolittoral zone in Antarctic. Both compounds exhibited no cytotoxicity (K562, A549, HCT116, and HeLa cell lines) at IC_50_ > 10 μM, but compound **199** was slightly against *Proteusbacillus vulgaris* with an MIC value of 22.5 μM [68].

**Figure 34:**
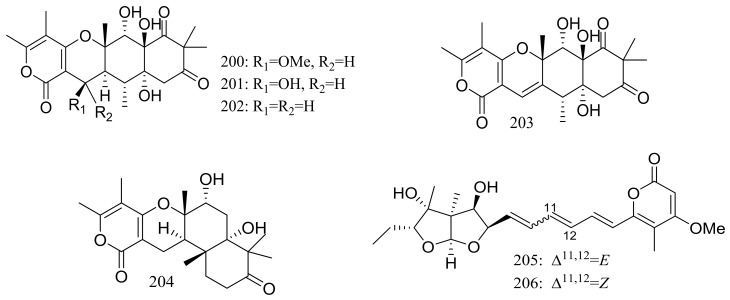
Novel natural products derived from psychrophilic fungi (compounds **200**–**206**).

The soil-derived fungus *Aspergillus ochraceopetaliformis* SCSIO 05702 collected near the Great Wall station (Chinese Antarctic station) yielded five new highly oxygenated *α*-pyrone merosesquiterpenoids named ochraceopones A–E (**200**–**204**), the known asteltoxin (**205**), and a new double bond isomer of asteltoxin named isoasteltoxin (**206**) (Figure 34). Compounds **200**–**203** possessed a linear tetracyclic carbon skeleton, which was distinguished from the reported angular tetracycle structure. Compounds **200**, **205**, and **206** displayed antiviral activity against the H1N1 and H3N2 influenza viruses with IC_50_ values of >20.0/12.2, 0.54/0.84, and 0.23/0.66 μM, respectively (the positive control tamiflu IC_50_ 16.9/18.5 nM). In addition, the selectivity indexes (SI = CC_50_/IC_50_) of anti-H1N1 activity of **205** (SI = 0.44) and **206** (SI = 2.35) suggested that the geometry of the ∆^11^ double bond in the polyene chain might accelerate the anti-H1N1 activity and selectivity index [69].

**Figure 35:**
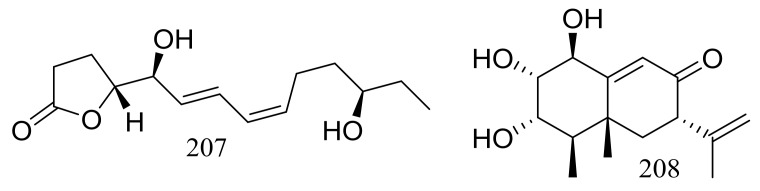
Novel natural products derived from psychrophilic fungi (compounds **207**–**208**).

A furanone derivative, butanolide A (**207**) and a sesquiterpene, guignarderemophilane F (**208**) (Figure 35) were produced by the Antarctic seabed sediment-derived fungus *Penicillium* sp. S-1-18 via the bioassay guidance. Compound **207** could moderately inhibited protein tyrosine phosphatase 1B (PTP1B) with an IC_50_ value of 27.4 μM [70].

**Figure 36:**
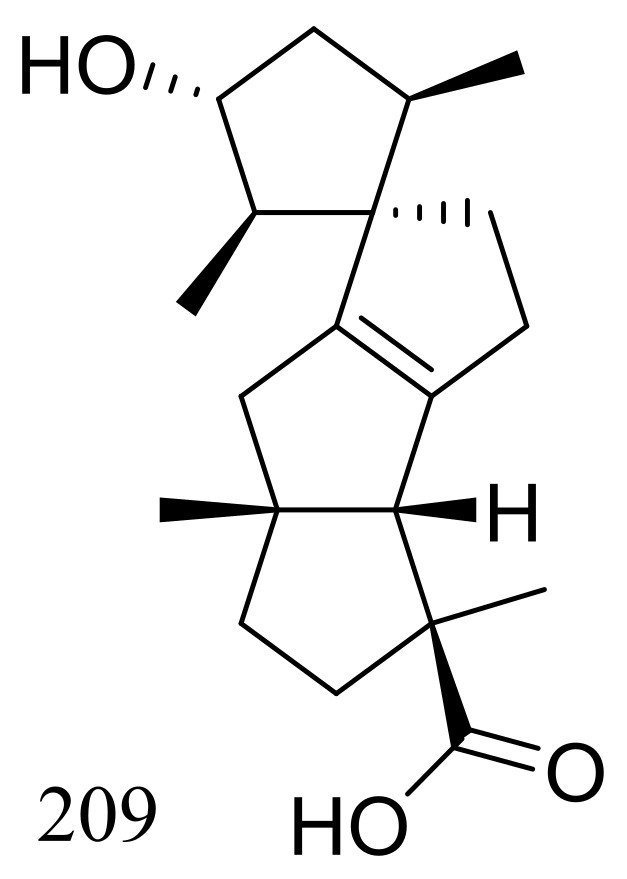
Novel natural products derived from psychrophilic fungi (compound **209**).

*Penicillium granulatum* MCCC 3A00475 obtained from the Prydz Bay of Antarctica yielded an unusual spirocyclic diterpene named spirograterpene A (**209**) (Figure 36). Antiallergic effect was tested in immunoglobulin E-mediated rat mast RBL-2H3 cells and compound **209** was just little weaker active than loratadine at 20 μg/mL [71].

## 4. Thermophilic Fungi

**Figure 37:**
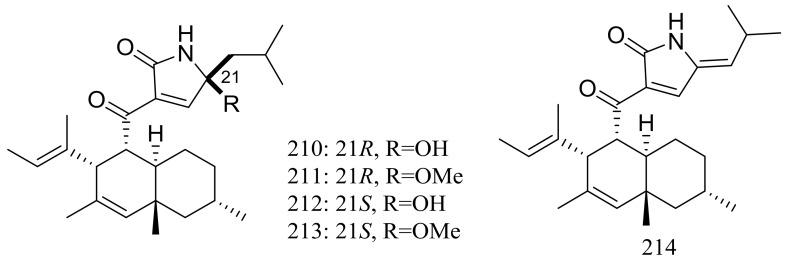
Novel natural products derived from thermophilic fungi (compounds **210**–**214**).

Five new polyketides (**210**–**214**) (Figure 37) were produced by *Myceliophthora thermophila* obtained from the soil of fumaroles in Taiwan. Compounds **210**–**212** showed cytotoxic activity against A549, Hep3B, MCF-7 and HepG2 cell lines with IC_50_ values ranging from 0.25 to 1.30 μg/mL [72].

**Figure 38:**
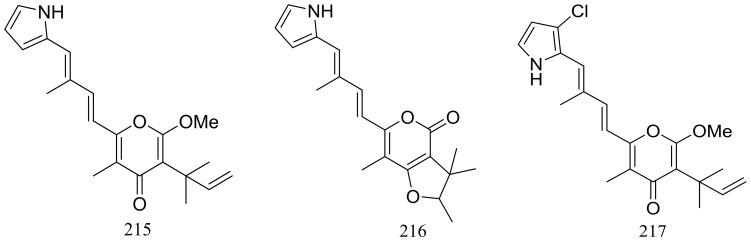

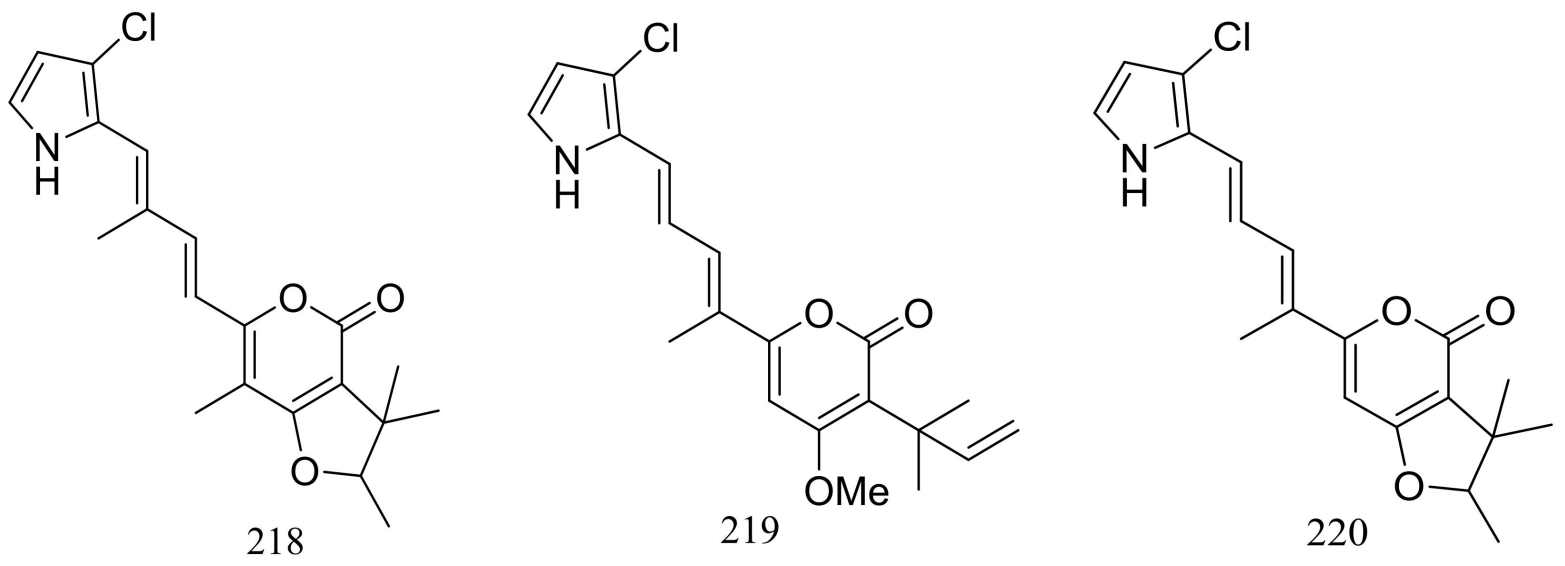
Novel natural products derived from thermophilic fungi (compounds **215**–**220**).

The EtOAc extract of the mass mycelium and PDA media of *Malbranchea sulfurea* which was obtained from the soil of fumaroles in Sihchong River Hot Spring Zone, displayed strong cytotoxicity against several cancer cell lines. Further bioassay-guided fractionation and chromatographic separation of the extract led to the isolation of six photosensitive polyketides named malbranpyrroles A–F (**215**–**220**) (Figure 38). Cytotoxic activities against PANC-1, HepG2 and MCF-7 cancer cell lines were tested and IC_50_ values for compounds **217**–**220** ranged from 3 to 11 μM. Flow cytometric measurement for cell cycle analysis showed that when treated by the malbranpyrroles the percentage of MCF-7 and HepG2 cells in G0/G1 phase was slightly increased, and the results suggested that these cytotoxic compounds could arrest the two cancer cell lines at G0 phase via inhibiting some cellular signaling pathways. According to the structure-bioactivity relations, the chlorine atom might be the pharmacophore for cytotoxicity [73].

**Figure 39:**
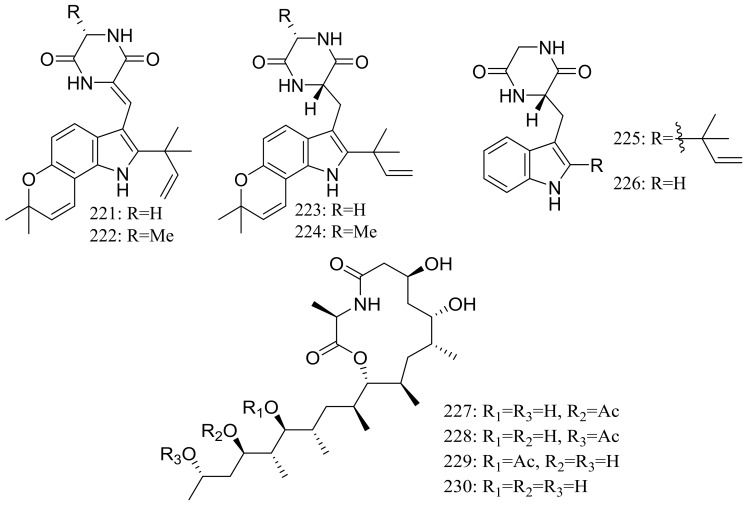

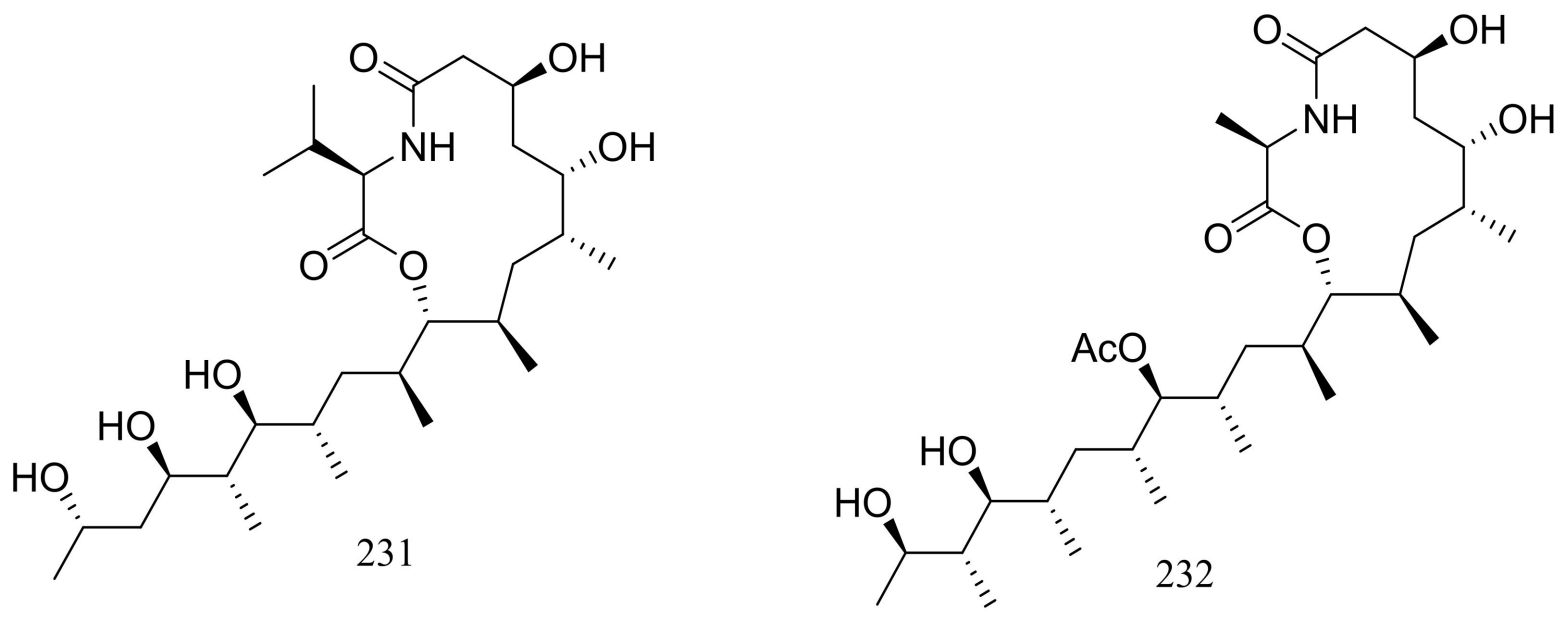
Novel natural products derived from thermophilic fungi (compounds **221**–**232**).

From two fungal strains *Talaromyces thermophilus* YM1-3 and YM3-4, both collected from Tengchong hot springs, six new indole alkaloids including talathermophilins A–B (**221**–**222**) (Figure 39) [74], two analogues of notoamide E (**223** and **224**), one analogue of preechinulin (**225**), and a natural occurring cyclo (glycyltryptophyl) (**226**) (Figure 39) [75], as well as a novel class of PKS-NRPS hybrid molecules named thermolides A–F (**226**–**232**) (Figure 39) [76] have been described. Compounds **221** and **222** exhibited moderate nematicidal activities against the worms of the free-living nematode *Panagrellus redivivus* with rates of 38% and 44% at 400 μg/mL for 72 h respectively. Compounds **227**–**232** possessed a 13-membered lactam-bearing macrolactone but only **227** and **228** displayed potent nematicidal activities against three notorious nematodes (*Meloidogyne incognita*, *Bursaphelenches siylopilus*, and *Panagrellus redivivus*) with LC_50_ values between 0.5 and 1 μg/mL. No information on the biological activities of compounds **223**–**226** was given.

**Figure 40:**
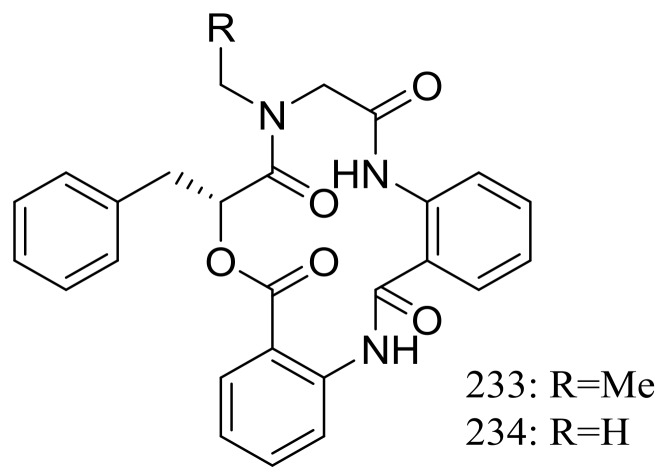
Novel natural products derived from thermophilic fungi (compounds **233**–**234**).

Clavatustides A–B (**233**–**234**) (Figure 40) containing an unusual anthranilic acid dimer and a D-phenyllactic acid residues were produced by *Aspergillus clavatus* C2WU isolated from the crab *Xenograpsus testudinatus*, which lived at extreme, toxic habitat around the sulphur-rich hydrothermal vents in Taiwan Kueishantao. The two novel cyclodepsipeptides significantly suppressed the proliferation of HepG2 cells in a dose-dependent manner, and cell cycle analysis suggested that **233** and **234** could induce G1 arrest and inhibit G1/S phase transition [77].

**Figure 41:**
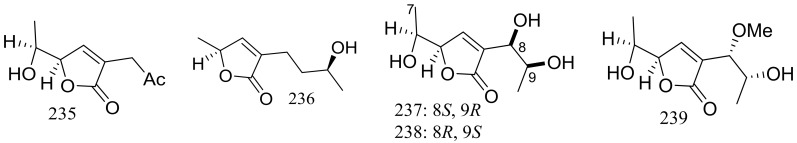

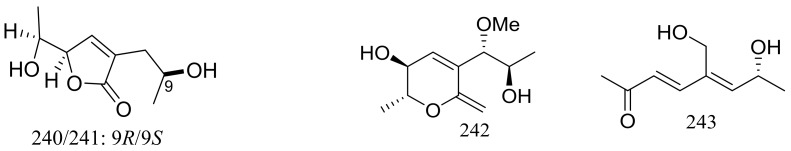
Novel natural products derived from thermophilic fungi (compounds **235**–**243**).

Nine new C_9_ polyketides named aspiketolactonol (**235**), aspilactonols A–F (**236**–**241**), aspyronol (**242**) and epiaspinonediol (**243**) (Figure 41) have been described as metabolites of *Aspergillus* sp. 16-02-1, which was collected at a Lau Basin hydrothermal vent (depth 2255 m, 114 °C) in the Southwest Pacific. Compounds **240** and **241** were obtained as a mixture in a diastereomeric ratio of 1:1. The possible biosynthetic pathways for all compounds were proposed and discussed. The cytotoxic activities (IC_50_ value) against HL-60 cells of compounds **242** and **243** were 241.2 and 192.9 μM respectively. For compounds **235**–**241** very weak cytotoxic activities (K562, HL-60, HeLa, or BGC-823) were observed with inhibitory rates less than 20% at 100 μg/mL [78].

**Figure 42:**
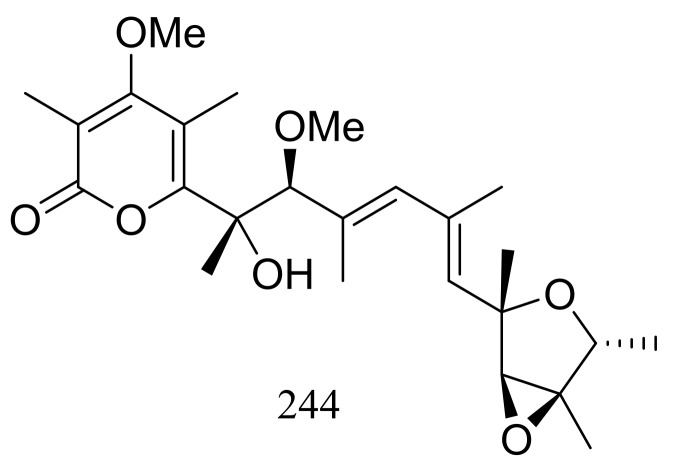
Novel natural products derived from thermophilic fungi (compound **244**).

A hydrothermal vent fungus *Penicillium* sp. Y-50-10 collected from the sulfur rich sediment (Kueishantao, Taiwan) yielded methyl isoverrucosidinol (**244**) (Figure 42). This new verrucosidin derivative displayed weak antibiotic activity against *Bacillus subtilis* with an MIC value of 32 μg/mL [79].

**Figure 43:**
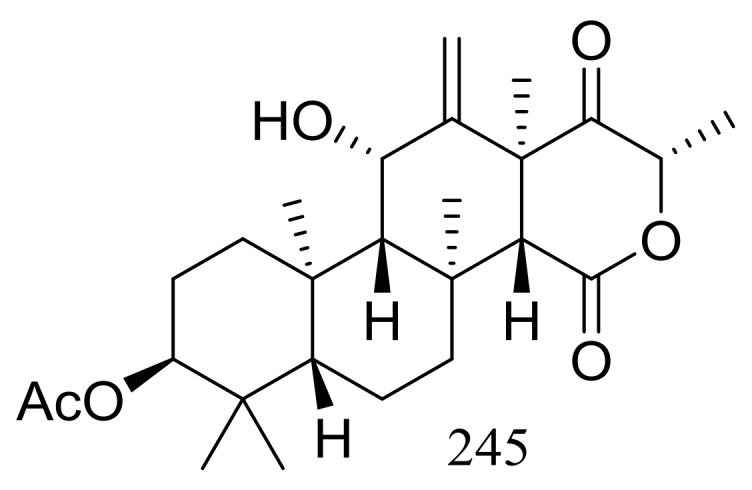
Novel natural products derived from thermophilic fungi (compound **245**).

The soil-derived thermophilic fungal strain *Aspergillus terreus* TM8 collected from a hot desert place (~50 °C) in South Egypt produced a new highly oxygenated tetracyclic meroterpenoid, terretonin M (**245**) (Figure 43). The crude extract of the mass mycelium and solid rice meida could slightly inhibit the growth of *Proteus* sp., *Candida albicans,* and *Streptococcus pyogenes*, while authors failed to isolate the active ingredient [80].

## 5. Halophilic Fungi

**Figure 44:**
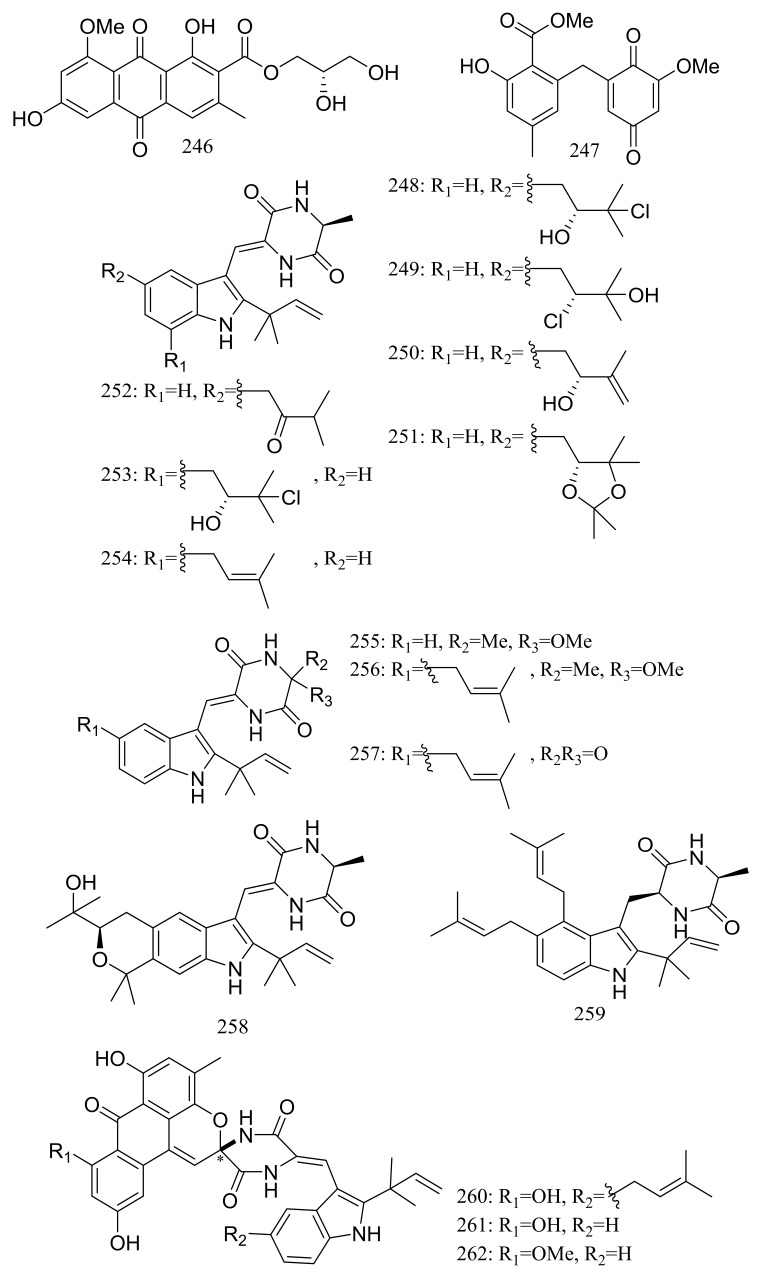
Novel natural products derived from halophilic fungi (compounds **246**–**262**). * Absolute configuration is not determined.

Diverse novel compounds have been described from the halotolerant fungal strain *Aspergillus variecolor* B-17, which was isolated from the sediments collected in Jilantai salt field, Alashan, Inner Mongolia, China. Variecolorquinones A–B (**246**–**247**) (Figure 44) are two new quinone type compounds with cytotoxic activities against A549 cells (compound **246,** IC_50_ 3.0 μM), HL60 cells (compound **247**, IC_50_ 1.3 μM) and P388 cells (compound **247**, IC_50_ 3.7 μM) [81]. Variecolorins A–L (**248**–**259**) (Figure 44) exhibited no cytotoxicity (P388, HL-60, BEL-7402, and A-549 cell lines) but A–K (**248**–**258**) showed weak radical-scavenging activity against DPPH with IC_50_ values ranging from 75 to 102 μM [82]. Variecolortides A–C (**260**–**262**) (Figure 44) shared an unprecedented ‘spiro-anthronopyranoid diketopiperazine’ structure with a stable hemiaminal function. All three compounds showed weak cytotoxic activity against K-562 cell line with IC_50_ values of 61, 69 and 71 μM respectively (the positive control paclitaxel IC_50_ 0.93 μM) and showed very slight radical-scavenging activity against DPPH radical with IC_50_ values of 63, 84 and 91 μM, respectively (the positive control vitamin C IC_50_ 22 μM) [83].

**Figure 45:**
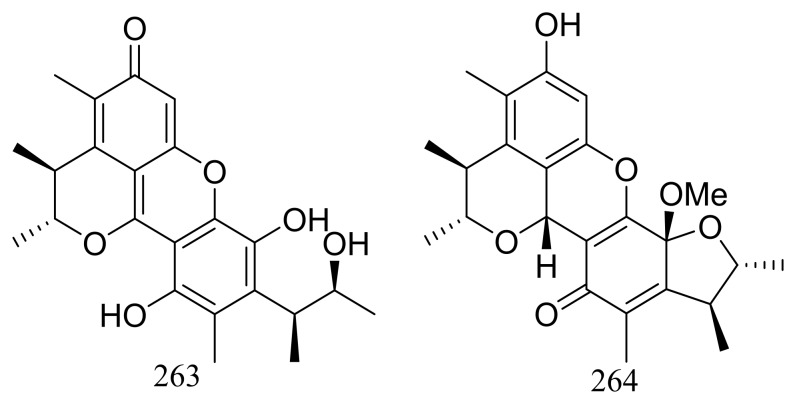
Novel natural products derived from halophilic fungi (compounds **263**–**264**).

Pennicitrinone C (**263**) and penicitrinol B (**264**) (Figure 45), two new citrinin dimers were produced by the halotolerant fungal strain *Penicillium citrinum* B-57 obtained from the sediments in Jilantai salt field, Alashan, Inner Mongolia, China. Compound **263** scavenged DPPH radicals with IC_50_ value of 55.3 μM (the positive control L-ascorbic acid IC_50_ 22.7 μM) but exhibited no cytotoxic activity against P388, A549, BEL7402 or HL60 cell lines (IC_50_ > 50 μM) [84].

**Figure 46:**
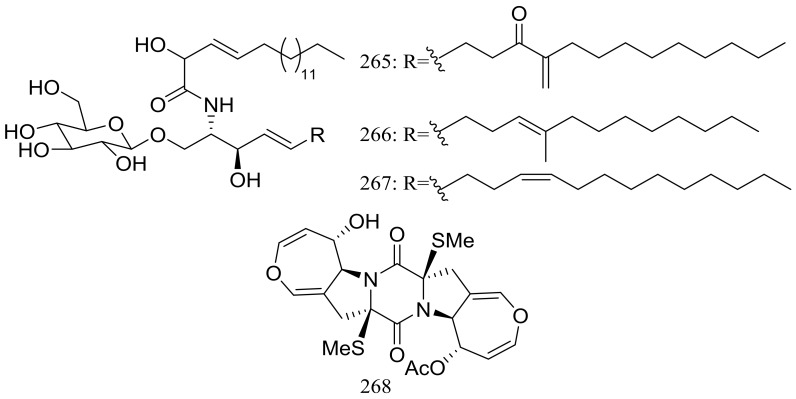
Novel natural products derived from halophilic fungi (compounds **265**–**268**).

Three new cerebrosides, alternarosides A–C (**265**–**267**) and one new diketopiperazine alkaloid, alternarosin A (**268**) (Figure 46) were produced by the halotolerant fungus *Alternaria raphani* THW-18, which was obtained from a sediment sample in the Hongdao sea salt field, China. Antimicrobial activities against *Escherichia coli*, *Bacillus subtilis*, and *Candida albicans* were evaluated and MIC values for four compounds ranged from 70 to 400 μM. Neither cytotoxicity (P388, HL-60, A549, and BEL-7402 cell lines) nor DPPH radical-scavenging activity was detected [85].

**Figure 47:**
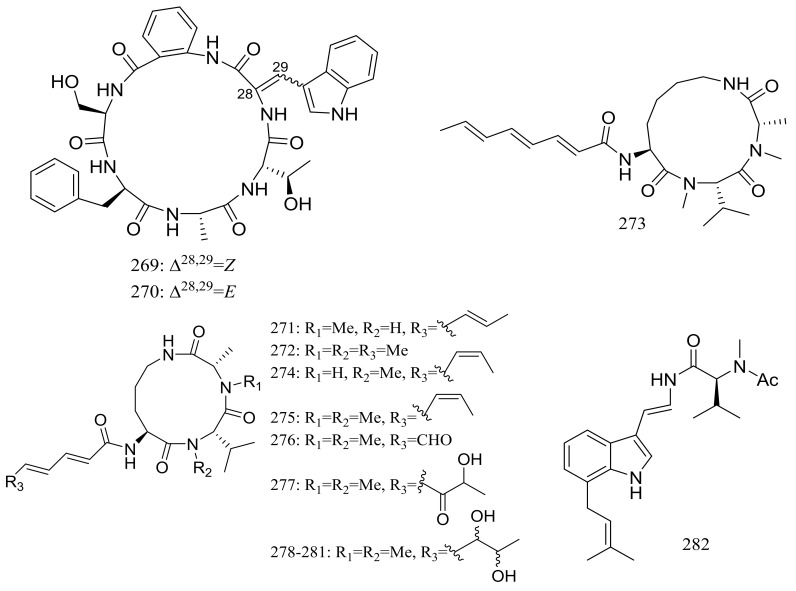
Novel natural products derived from halophilic fungi (compounds **269**–**282**).

Sclerotides A–B (**269**–**270**) (Figure 47) were novel cyclic hexapeptides produced by the halotolerant *Aspergillus sclerotiorum* PT06-1 (the Putian Sea Salt Field, China) in a nutrient-limited hypersaline medium. In the general bioactivity profiling programs including cytotoxic and antimicrobial testing, both compounds inhibited *Candida albicans* with MIC values of 7.0 and 3.5 μM respectively. Besides, compound **270** displayed weak cytotoxic activity against HL-60 cells (IC_50_ 56.1 μM) and antibacterial activity against *Pseudomonas aeruginosa* (MIC 35.3 μM) [86]. The same research group subsequently obtained eleven new aspochracin-type cyclic tripeptides named sclerotiotides A–K (**271**–**281**) (Figure 47) [87] and one new cytotoxic indole-3-ethenamide (**282**) (Figure 47) [88] from the same halotolerant fungal strain in a nutrient-rich hypersaline medium. Compounds **278**–**281** were four isomers with the same molecular formula, and the NMR data suggested that **278**/**280** and **279**/**281** were enantiotopic in the fatty acid moiety respectively. When evaluated for antimicrobial and cytotoxic activities compounds **271**, **272**, **276**, and **279** exhibited antifungal activities against *Candida albicans* with MIC values of 7.5, 3.8, 30, and 6.7 μM respectively, while compound **282** showed cytotoxic activity against A-549 and HL-60 cells with IC_50_ values of 3.0 and 27 μM respectively.

## 6. Xerophilic Fungi

**Figure 48:**
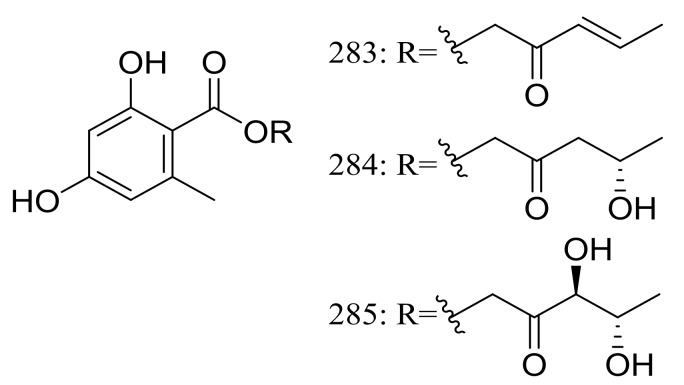
Novel natural products derived from xerophilic fungi (compounds **283**–**285**).

Globosumones A–C (**283**–**285**) (Figure 48) are three new esters of orsellinic acid isolated from *Chaetomium globosum* endophytic on *Ephedra fasciculata* (Mormon tea), which was collected from the Sonoran Desert. Cytotoxic activities against four cancer cell lines (NCI-H460, MCF-7, SF-268, and MIA Pa Ca-2) were tested and only compounds **283** and **284** were moderately active with IC_50_ values of 6.5 to 30.2 μM (the positive control doxorubicin IC_50_ 0.01 to 0.07 μM) [89].

**Figure 49:**
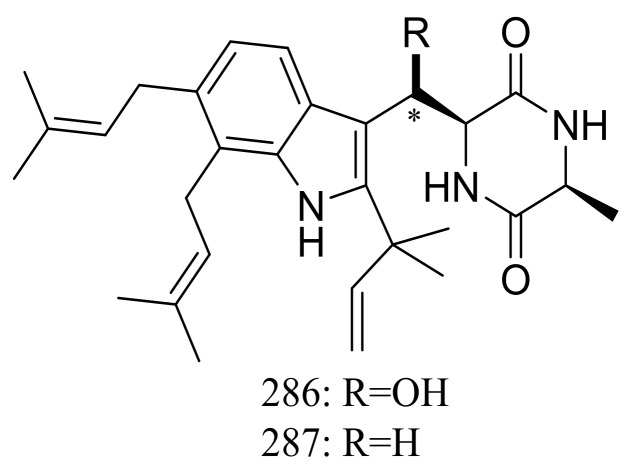
Novel natural products derived from xerophilic fungi (compounds **286**–**287**). * Absolute configuration is not determined.

The xerophilic fungus *Aspergillus restrictus* A-17 obtained from house dust yielded two new dioxopiperazine derivatives, arestrictins A–B (**286**–**287**) (Figure 49). The biological activity of them was not tested [90].

**Figure 50:**
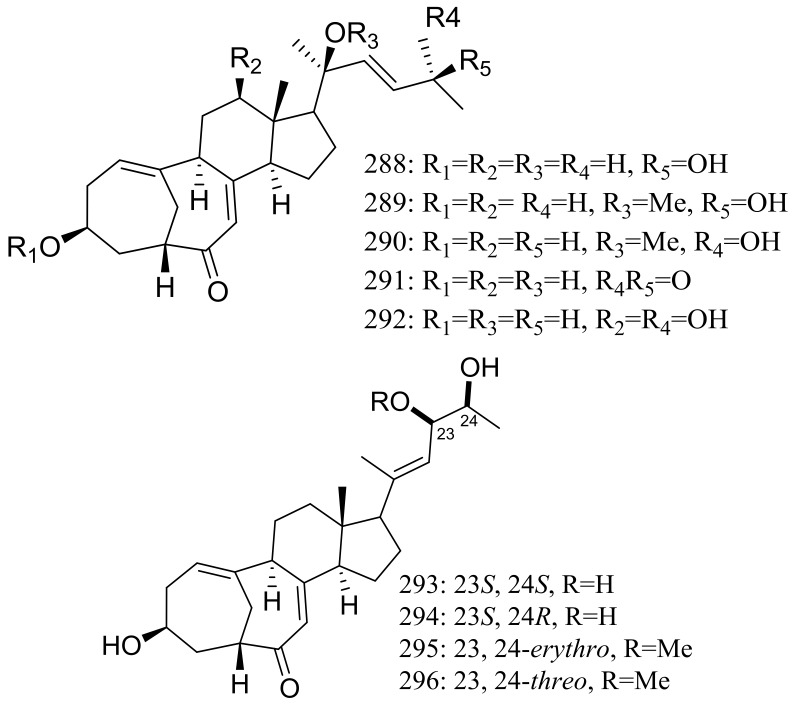

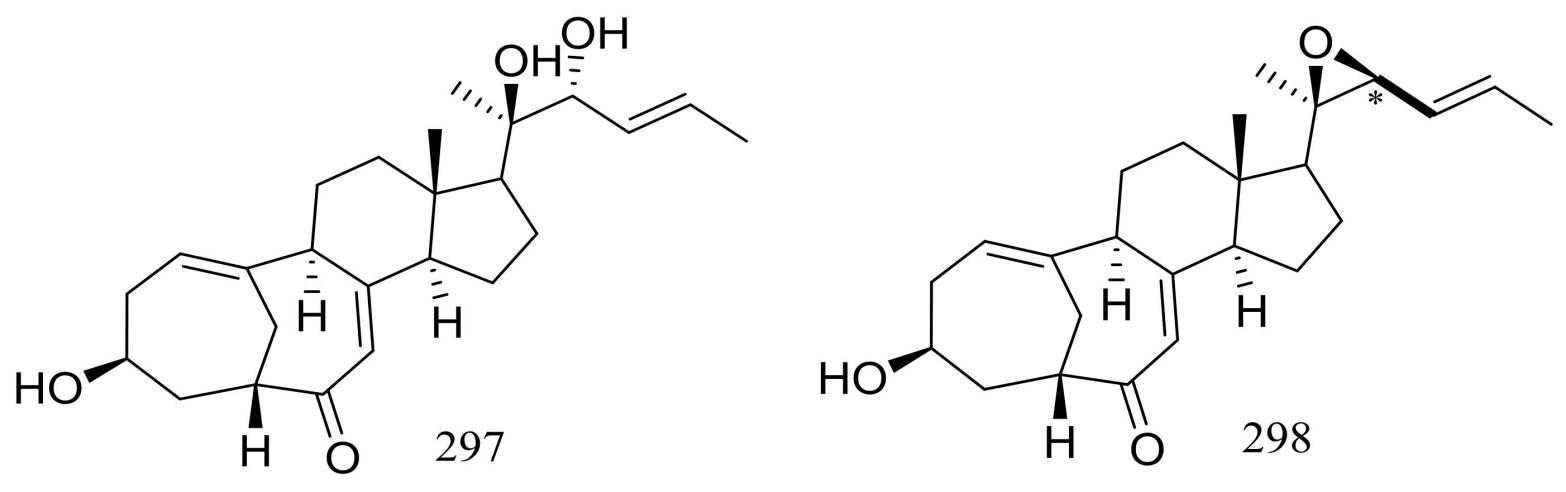
Novel natural products derived from xerophilic fungi (compounds **288**–**298**). * Absolute configuration is not determined.

The culture broth of the volcanic ash-derived fungus *Penicillium citrinum* HGY1-5 collected from the extinct volcano Huguangyan in Guangdong, China, afforded eleven new unusual C25 steroid isomers with bicyclo[4.4.1]A/B rings named 24-*epi*-cyclocitrinol (**288**), 20-*O*-methyl-24-*epi*cyclocitrinol (**289**), 20-*O*-methylcyclocitrinol (**290**), 24-oxocyclocitrinol (**291**), 12*R*-hydroxycyclocitrinol (**292**), neocyclocitrinols B and D (**293** and **294**), *erythro*-23-*O*-methylneocyclocitrinol (**295**), *threo*-23-*O*-methylneocyclocitrinol (**296**), isocyclocitrinol B (**297**), and precyclocitrinol B (**298**) (Figure 50). The evaluation for biological activity of all steroids with the cAMP assay in GPR12-CHO and WT-CHO cells indicated that compounds **288**, **293** and **296** could induce the production of cAMP in GPR12-transfected CHO cells at 10 μM [91].

## 7. Acidophilic or Alkaliphilic Fungi

**Figure 51:**
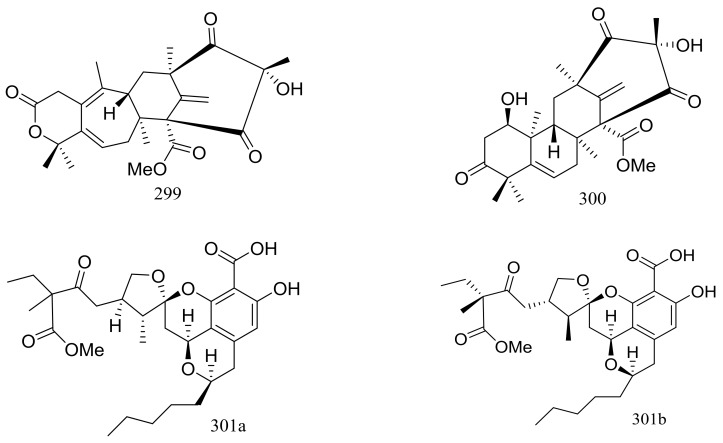

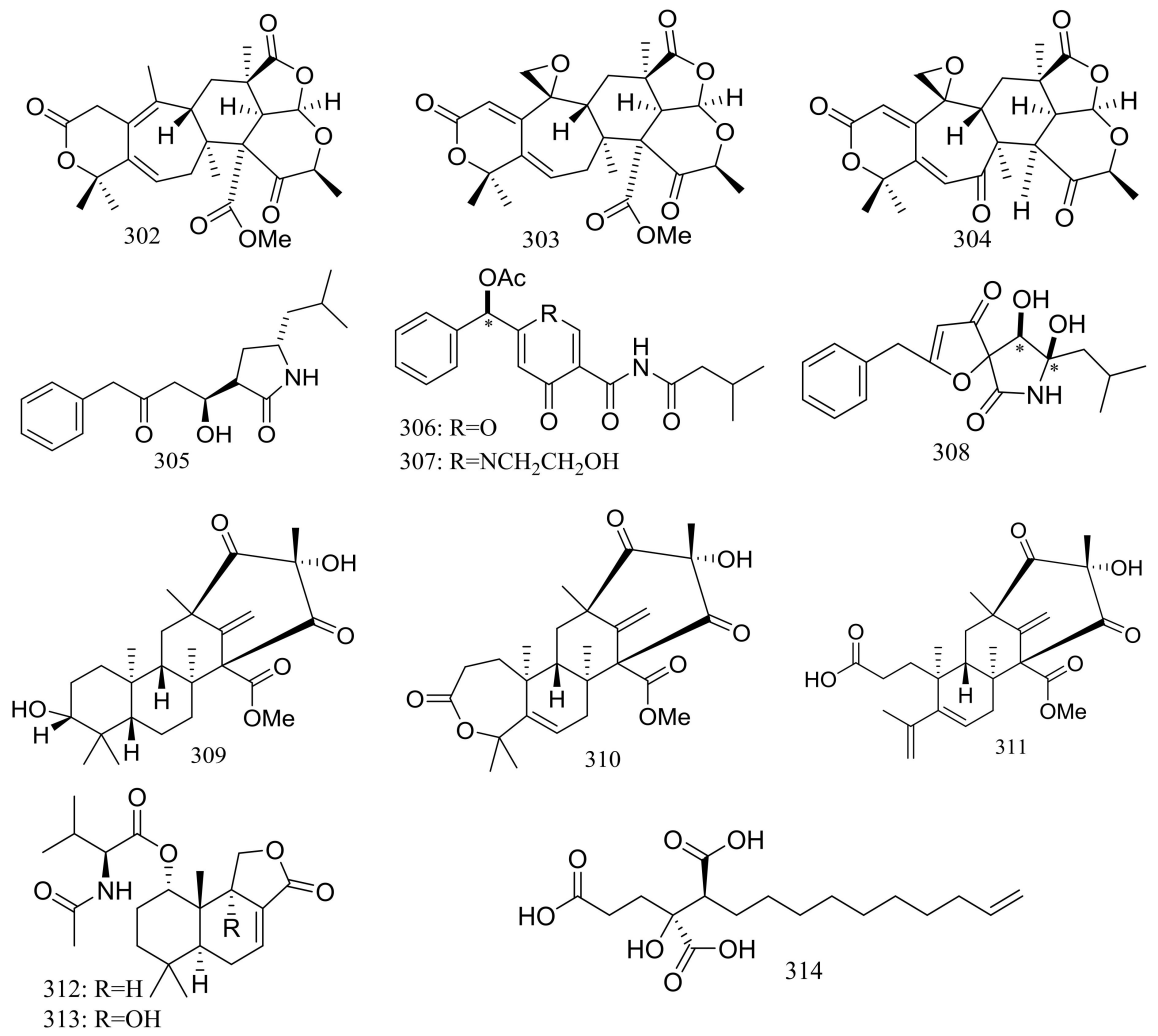
Novel natural products derived from acidophilic or alkaliphilic fungi (compounds **299**–**314**). * Absolute configuration is not determined.

Since 2004, several new compounds have been obtained from *Penicillium* species growing in the Berkeley Pit Lake (Butte, Montana), which is an abandoned open-pit copper mine filled with 30 billion gallons of acidic, metal-contaminated water. Two novel hybrid polyketide-terpenoids named berkeleydione (**299**) and berkeleytrione (**300**) (Figure 51) [92], one novel spiroketal named berkelic acid (**301a**) (Figure 51) [93], as well as berkeleyacetals A–C (**302**–**304**) (Figure 51) [94] were isolated from an unidentified *Penicillium* species. In 2018 Fürstner group revised the absolute configuration of berkelic acid (**301b**) through an elegant synthetic, NMR, and crystallographic study [95]. Compounds **299**–**304** were found to be inhibitors of matrix metalloproteinase-3 (MMP-3) and the cysteine protease caspase-1 (Casp-1) in the micromolar or millimolar range. In addition, compounds **299** and **304** displayed cytotoxic activity against NCI-H460 cells (GI_50_ 0.398 μM), while **301** against OVCAR-3 cells (GI_50_ 0.091 μM). Berkeleyamides A–D (**305**–**308**) (Figure 51) [96] and berkeleyones A–C (**309**–**311**) (Figure 51) [97] were isolated from *Penicillium rubrum*. Compounds **305**–**308** were able to suppress caspase-1 and MMP-3 in the low micromolar range. Effects on inhibiting the production of interleukin 1-*β* in THP-1 cells was tested and IC_50_ values for compounds **309** and **310** were 2.7 and 3.7 μM respectively (the positive control Ac-YVAD-CHO IC_50_ 2.0 μM). Two new drimane sesquiterpene lactones named berkedrimanes A–B (**312**–**313**) and one new tricarboxylic acid derivative (**314**) (Figure 51) were produced by *Penicillium solitum*. Compounds **312** and **313** inhibited caspase-1 and caspase-3 in the micromolar range and mitigated the production of IL-1*β* by intact inflammasomes at low micromolar concentrations [98].

## 8. Conclusions

In this review, a total of 314 novel compounds (161 bioactive ones) from extremophilic fungi have been compiled, including 58 terpenoids/steroids, 130 alkaloids/peptides/amides, 50 quinones/phenols, 14 esters/lactones, 11 xanthones, 31 polyketides, and 20 other structure compounds. All compounds were obtained from 56 fungal strains, most of which were asexual stages of ascomycetes e.g., *Penicillium* sp. (21 strains), *Aspergillus* sp. (11 strains), and other species (22 strains). Only one basidiomycete (*Acaromyces* sp.) and one zygomycete (*Malbranchea* sp.) appeared in the present review.

As demonstrated by this review, fungi from extreme environments are a rich source for novel natural products, even though the research on them is not as up-to-date as the research on fungi in other mesophilic environments due to the difficulties in both sample collection and cultivation. However, with the fast development of modern instruments and techniques in the post-genomic era, some groups have obtained many new compounds from one strain by changing its cultivation conditions or creating a mutant, which significantly contributes to make full use of these precious biological resources.

## Figures and Tables

**Table 1:** Novel natural products isolated from extremophilic fungi.

The Type of Compound	Compounds	Biological Activity	References
***Terpenoids and steroids***	23, 24 *, 25–28	Cytotoxic	[25]
	(39–41) *, 42, 43 *, 44, 45	Cytotoxic and/or antiviral	[29,30]
	(145–150) *, 154, 155 *, 156, 157 *	Cytotoxic and/or antimicrobial	[52,53,56,57]
	176 *, 177–179, 180 *, 181–183, 184 *	Cytotoxic	[62,63]
	**190, 191**		[66]
	200 *, 201–204	Antiviral	[69]
	**208**		[70]
	**209**	Antiallergic	[71]
	**245**		[80]
	288 *, 289–292, 293 *, 294, 295, 296 *, 297, 298	Induce cAMP production	[91]
	299 *, 300 *	Inhibit MMP-3 and Casp-1 and/or cytotoxic	[92]
	309 *, 310 *, 311	Mitigate IL-1*β* production	[97]
***Alkaloids, peptides, and amides ***	10, 11 *, 12, 13, 14 *	Anti-inflammatory	[24]
	15 *, 16, 17 *, 18 *, 19–22	Cytotoxic	[25,26]
	**29–33**		[27]
	(47–55) *	Insecticidal	[32]
	**73**		[36]
	74 *, 75, 76	Inhibit α-glucosidase	[37]
	86 *, 87 *, 88, 89	Antimicrobial	[40]
	**104, 105**		[43]
	**108, 112**		[44]
	(124–135) *, 136–138, (139–142) *	Antiviral or antimicrobial	[48,49]
	144 *	Cytotoxic	[51]
	151 *, 152, 153	Cytotoxic	[55]
	(167–169) *, 170–173	Cytotoxic	[60]
	**185**		[63]
	**187–189**		[65]
	192 *, 193 *, 194, 195, 196 *, 197	Reduce intracellular lipid accumulation	[67]
	198, 199 *	Antimicrobial	[68]
	221 *, 222 *, 223–226	Nematicidal	[74,75]
	233 *, 234 *	Cytotoxic	[77]
	(248–258) *, 259, (260–262) *	Radical-scavenging and/or cytotoxic	[82,83]
	(265–268) *	Antimicrobial	[85]
	(269–272) *, 273–275, 276 *, 277, 278, 279 *, 280, 281, 282 *	Antimicrobial and/or cytotoxic	[86,87,88]
	**286, 287**		[90]
	(305–308) *	Inhibit MMP-3 and Casp-1	[96]
	312 *, 313 *	Inhibit MMP-3 and Casp-1, and mitigate IL-1*β* production	[98]
***Quinones and phenols ***	**46**		[31]
	**56**		[33]
	63–67, 68 *, 69–71	Antimicrobial and cytotoxic	[35]
	**77**	Cytotoxic	[38]
	79 *, 80 *, 81, 82 *, 83, 84 *, 85 *	Cytotoxic	[39]
	94–97, 98 *, 99	Activate Nrf2	[41]
	106 *, 107	Antilarval	[43]
	109, 110 *, 111 *	Inhibit BRD4	[44]
	**114–119**		[46]
	143 *	Antimicrobial	[50]
	158–160, 161 *, 162 *, 163–166	Antimicrobial	[58,59]
	246 *, 247 *	Cytotoxic	[81]
	263 *, 264	Radical-scavenging	[84]
***Esters and lactones ***	**78**		[38]
	207 *	Inhibit PTP1B	[70]
	227 *, 228 *, 229–232	Nematicidal	[76]
	283 *, 284 *, 285	Cytotoxic	[89]
	(302–304) *	Cytotoxic and/or inhibit MMP-3	[94]
***Xanthones ***	57 *, 58, 59 *, 60 *	Antimicrobial	[34]
	**61, 62**		[35]
	**72**		[36]
	**90–93**		[40]
***Polyketides ***	100, 101, 102 *, 103 *	Cytotoxic	[42]
	120–122, 123 *	Cytotoxic	[47]
	174 *, 175	Inhibit NF-κB	[61]
	186 *	Antimicrobial	[64]
	(210–212) *, 213, 214	Cytotoxic	[72]
	215, 216, (217–220) *	Cytotoxic	[73]
	235–241, 242 *, 243 *	Cytotoxic	[78]
***Others***	(1–9) *	Cytotoxic	[20,21,22,23]
	(34–38) *	Antimicrobial	[28]
	113 *	Antimicrobial	[45]
	205 *, 206 *	Antiviral	[69]
	244 *	Antimicrobial	[79]
	301 *	Inhibit MMP-3 and Casp-1	[93]
	**314**		[98]

* bioactive compounds.
